# A Fat-Facets-Dscam1-JNK Pathway Enhances Axonal Growth in Development and after Injury

**DOI:** 10.3389/fncel.2017.00416

**Published:** 2018-02-08

**Authors:** Marta Koch, Maya Nicolas, Marlen Zschaetzsch, Natalie de Geest, Annelies Claeys, Jiekun Yan, Matthew J. Morgan, Maria-Luise Erfurth, Matthew Holt, Dietmar Schmucker, Bassem A. Hassan

**Affiliations:** ^1^Laboratory of Neurogenetics, Center for Brain and Disease Research, Vlaams Instituut voor Biotechnologie (VIB), Leuven, Belgium; ^2^Center for Human Genetics, University of Leuven School of Medicine, KU Leuven, Leuven, Belgium; ^3^Neuronal Wiring Lab, Center for Brain and Disease Research, Vlaams Instituut voor Biotechnologie (VIB), Leuven, Belgium; ^4^Laboratory of Glia Biology, Center for Brain and Disease Research, Vlaams Instituut voor Biotechnologie (VIB), Leuven, Belgium; ^5^Centre National de la Recherche Scientifique, Institut National de la Santé et de la Recherche Médicale, Institut du Cerveau et de la Moelle Epinière, Hôpital Pitié-Salpêtrière, UPMC, Sorbonne Universités, Paris, France

**Keywords:** axonal growth, axonal injury, post-transcriptional reguylatiopn, Central nervous system, *Drosophila melanogaster*

## Abstract

Injury to the adult central nervous systems (CNS) can result in severe long-term disability because damaged CNS connections fail to regenerate after trauma. Identification of regulators that enhance the intrinsic growth capacity of severed axons is a first step to restore function. Here, we conducted a gain-of-function genetic screen in Drosophila to identify strong inducers of axonal growth after injury. We focus on a novel axis the Down Syndrome Cell Adhesion Molecule (Dscam1), the de-ubiquitinating enzyme Fat Facets (Faf)/Usp9x and the Jun N-Terminal Kinase (JNK) pathway transcription factor Kayak (Kay)/Fos. Genetic and biochemical analyses link these genes in a common signaling pathway whereby Faf stabilizes Dscam1 protein levels, by acting on the 3′-UTR of its mRNA, and Dscam1 acts upstream of the growth-promoting JNK signal. The mammalian homolog of Faf, Usp9x/FAM, shares both the regenerative and Dscam1 stabilizing activities, suggesting a conserved mechanism.

## Introduction

During CNS development axons grow in a tightly regulated manner to generate an intricate and complex pattern of neuronal connectivity. In most animal species, injury to the adult CNS, either by physical trauma or in the context of neurodegeneration, has devastating long-term consequences in part because of the inability of mature neurons to regenerate severed axons. Functional regeneration requires damaged axons to first start re-growing and then to continue to navigate through a strongly inhibitory environment, before they can reach their synaptic partners and establish functional connections. Both the presence of extrinsic inhibitory factors as well as a lack of intrinsic growth capacity prevent axonal regrowth in the injured CNS (Kaplan et al., 2015). However, targeting extrinsic inhibitory factors has so far led to limited regeneration of injured axons (Cafferty et al., 2010; Lee et al., 2010), suggesting that creating a permissive environment is not sufficient to allow regeneration. Even though neural circuits retain a remarkable degree of synaptic plasticity in adulthood, the mature CNS can no longer support the robust axonal growth that was once required to establish neuronal connectivity during development, suggesting that the neuronal intrinsic growth ability is largely lost. Indeed, mammalian CNS axons show a higher regenerative capacity during earlier stages of development, illustrating the importance of intrinsic factors to CNS regenerative failure (Shimizu et al., 1990; Liu et al., 2011). In PNS neurons, axonal injury results in a regeneration program that shares key molecular features with developmental axon growth (Makwana and Raivich, 2005; Harel and Strittmatter, 2006; Raivich and Makwana, 2007; Yaniv et al., 2012). In particular, the JNK pathway has emerged as a conserved signal for axonal growth and regeneration in the CNS and PNS in mammals, flies and worms (Raivich et al., 2004; Raivich and Makwana, 2007; Ayaz et al., 2008; Nix et al., 2011; Arthur-Farraj et al., 2012; Li et al., 2012). This suggests that conserved developmental axonal growth signaling pathways may be key targets to boost efficient regeneration after injury.

Studies in mice have made unique contributions to our understanding of the molecular basis of axonal regeneration. Nevertheless, the experiments are still costly and time-consuming and often necessitate a gene-by-gene approach. More recently, simpler genetic model organisms such *Caenorhabditis elegans* and *Drosophila* have proven useful to identify and study novel genes involved in axonal regrowth after injury (Yanik et al., 2004; Leyssen et al., 2005; Ayaz et al., 2008; Gabel et al., 2008; Chen et al., 2011; Kato et al., 2011; Fang and Bonini, 2012; Fang et al., 2012). Interestingly, unlike *C. elegans* neurons and developing *Drosophila* neurons, injured adult *Drosophila* CNS axons fail to regrow after injury, much like their mammalian counterparts (Ayaz et al., 2008). Furthermore, adult *Drosophila* CNS axons show remarkable morphological and genetic hallmarks of mammalian axonal responses to injury, including the formation of retraction bulbs, Wallerian degeneration of the distal fragment, transient upregulation of JNK, and regeneration upon activation of protein kinase A and JNK signaling (Leyssen et al., 2005; MacDonald et al., 2006; Ayaz et al., 2008). Another mediator of axonal injury responses in mouse models, the Dual Lucine Zipper Kinase/Wallenda (DLK1/Wnd) also plays similar roles when tested in *Drosophila* models of both axonal growth and injury. Interestingly, DLK1/Wnd activity has been linked to both cAMP and JNK signaling, suggesting a convergence of regenerative mechanisms (Itoh et al., 2009; Watkins et al., 2013; Valakh et al., 2015; Hao et al., 2016). This makes the *Drosophila* adult CNS a particularly powerful model system to systematically search for novel axonal regeneration genes.

Here, we performed a two-step genetic screen of ~300 genes selected by GO term, and identified 13 that promote axonal outgrowth during development in post-mitotic CNS neurons. We then tested those genes in an adult *Drosophila* model of CNS injury. Using this approach, we identified three robust axonal regeneration regulators, which we found to interact in a novel axonal growth and regeneration signaling pathway. Specifically, the deubiquitinating enzyme Fat facets (Faf) promotes axonal regrowth after injury via the Down syndrome cell adhesion molecule (Dscam1). Our findings suggest that Faf stabilizes Dscam1 by acting on Dscam1 3′-UTR through DLK1/Wnd and that Faf and Dscam1 act upstream of JNK signaling and its nuclear effector Kayak (Fos). The functional role of Faf in promoting axonal regeneration appears to be conserved in mammals, as suggested by the ability of the mouse homolog of Faf, Usp9X/FAM to also stabilize Dscam1 and promote axonal regrowth in the injured fly CNS.

## Results

### A genetic screen for axonal growth in development and after injury

**Figure 1:**
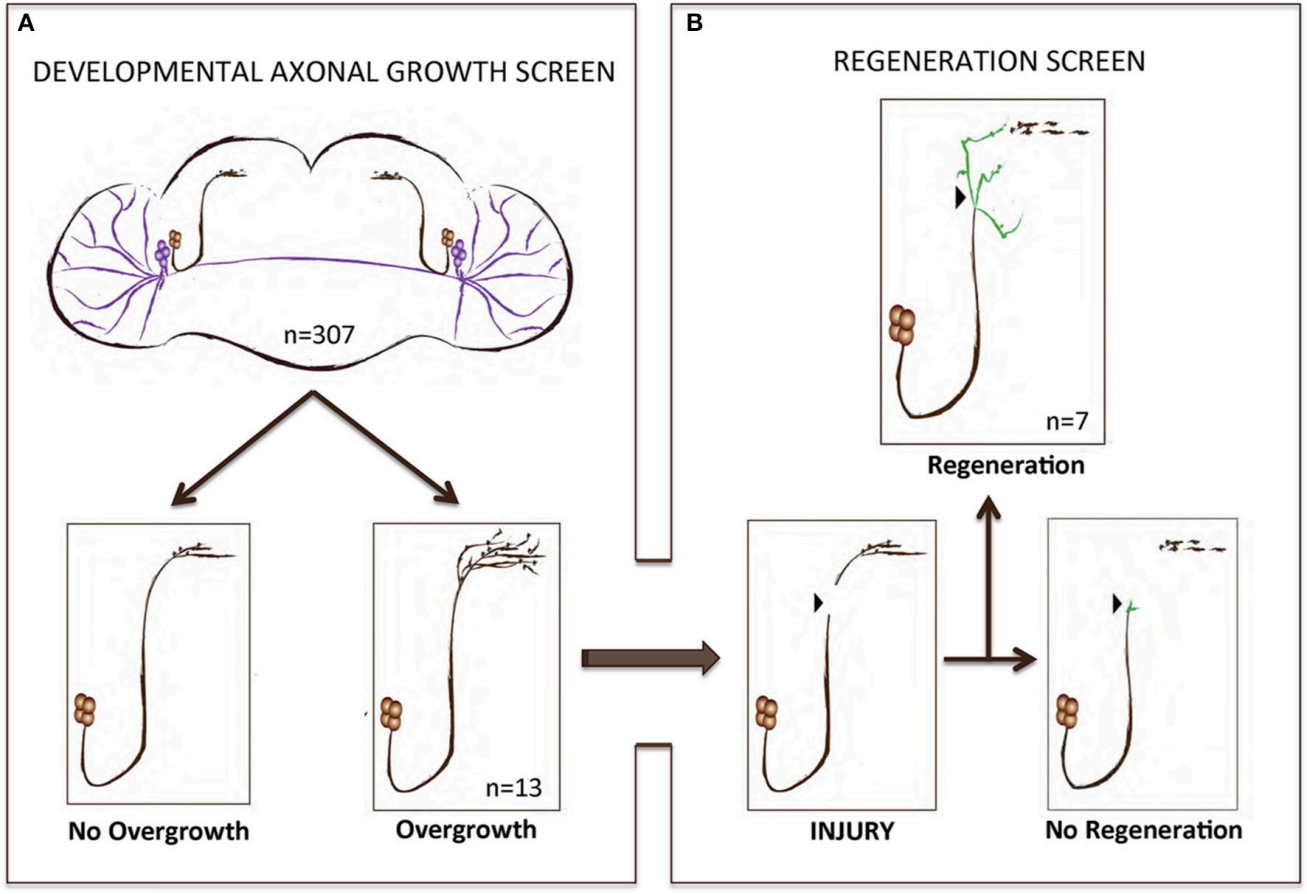
A gain-of-function screen for axonal growth in development and after injury. **(A,B)** Schematic illustrating the various outcomes for the development and injury steps of the screen. Both sLNVs (dark brown) and lLNVs (purple) are depicted in **(A)**. Gain-of-function the candidate genes specifically in PDF neurons appeared to increase growth in 4.2% (*n* = 13) of the cases. Genes that stimulated axonal growth were tested further in an injury paradigm in which the sLNV axonal projection is physically cut and sLNVs were accessed for regrowth 4 days post-injury **(B)**. Seven genes retained the ability to promote significant regeneration of injured axons.

**Figure 2:**
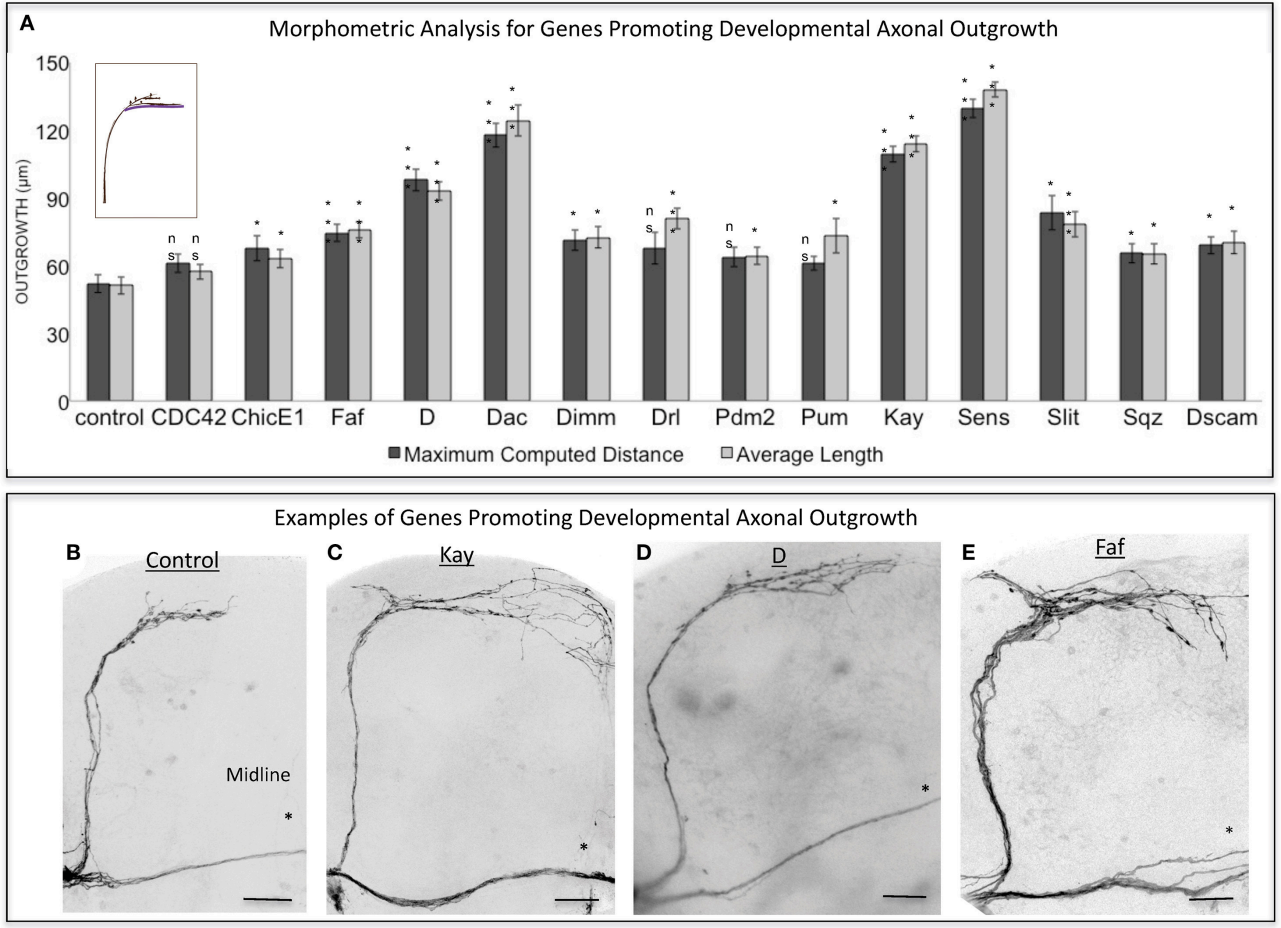
Analysis of axonal outgrowth in the developmental screen. **(A)** Morphometric analysis (Maximum Computed Distance and Average Length) of sLNv axonal projections where developmental overexpression of candidate genes has been specifically induced in the sLNvs. Axonal outgrowth is measured in μm. Purple trace in schematic represents measured axonal length. **(B–E)** Representative images of sLNv axonal arborization in wild type (control) adult flies **(B)** and in flies where developmental overexpression of *Kayak, kay* **(C)**, *Dichaete, D* **(D)** and *Fat Facets, faf* **(E)** has been specifically induced in the sLNvs. Scale bars are 20 μm. Genotype of flies in **(B)** is PDF-Gal4, UAS-GFP/+; PDF-Gal4, UAS-2x eGFP/+, in **(C)** is PDF-Gal4, UAS-GFP/+; PDF-Gal4, UAS-2x eGFP/+; UAS-Kay/+, in **(D)** is PDF-Gal4, UAS-GFP/+; PDF-Gal4, UAS-2x eGFP/+; UAS-D/+, in **(E)** is PDF-Gal4, UAS-GFP/+; PDF-Gal4, UAS-2x eGFP/+; UAS-Faf/+. Asterisk denotes the brain midline. ^*^p < 0.05; ^***^p < 0.001. n.s. indicates no statistical significance. Error bars represent SEM. Scale bars are 20 μm.

**Figure 3:**
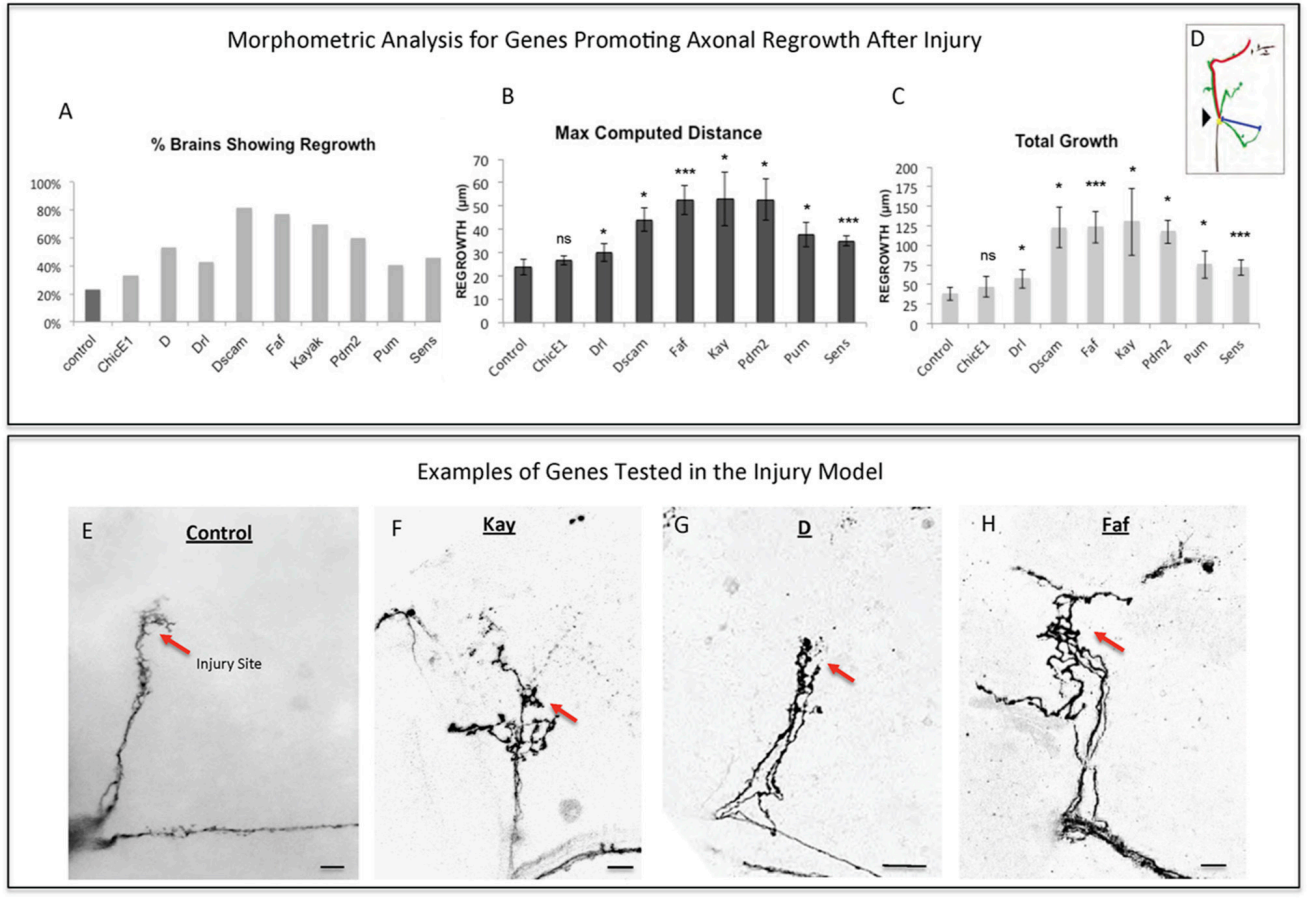
Analysis of axonal regrowth in the regeneration screen. **(A–D)** Analysis of axonal regrowth 4 days after injury. **(A)** Percentage of brains where at least one regenerated axonal sprout is detected (Capacity of regrowth). **(B,C)** Morphometric analysis [Maximum Computed Distance, **(B)** and Total Growth **(C)**] of regenerated sLNv axonal sprouts. Axonal regrowth is measured in μm. **(D)** Schematic simplifying how length of the regrown axonal sprouts is assessed. Yellow dot shows the point of injury; red trace represents maximum axonal length; blue trace represents axonal length measured in a straight line. **(E–H)** Representative images of sLNv axonal regrowth 4 days after injury in wild type (control) adult flies **(E)** and in flies where overexpression of *Kayak, kay* **(F)**, *Dichaete, D* **(G)**, and *Fat Facets, faf* **(I)** has been specifically induced in the sLNvs. Genotype of flies in **(E)** is PDF-Gal4, UAS-GFP/+; PDF-Gal4, UAS-2x eGFP/+, in **(F)** is PDF-Gal4, UAS-GFP/+; PDF-Gal4, UAS-2x eGFP/+; UAS-Kay/+, in **(G)** is PDF-Gal4, UAS-GFP/+; PDF-Gal4, UAS-2x eGFP/+; UAS-D/+, in **(H)** is PDF-Gal4, UAS-GFP/+; PDF-Gal4, UAS-2x eGFP/+; UAS-Faf/+. Red arrow denotes the injury point. ^*^p < 0.05; ^***^p < 0.001. n.s. indicates no statistical significance. Error bars represent SEM. Scale bars are 20 μm.

To perform a screen for axonal growth and regeneration (Figures 1A,B), we selected genes which: (1) are associated with the Gene Ontology (GO) terms neural development and neurite morphogenesis, (2) had Gal4 inducible transgenes available at the time of the initiation of the study and (3) represent a diversity of molecular functions, including receptors, protein turnover, transcription factors, and chromatin modifiers. Three hundred and seven genes matching these criteria were first tested for their ability to induce developmental axonal over-growth in small Lateral Neurons ventral (sLNv), a small cluster of neurons with a highly stereotyped axonal morphology which can be readily quantified (Leyssen et al., 2005; Helfrich-Förster et al., 2007) and that has been previously used to investigate the molecular mechanisms underlying regeneration in the fly CNS (Ayaz et al., 2008; Figures 1A, 2A,B). The post-mitotic *Pdf-Gal4* driver was used to express GFP together with each of the selected genes, and the length of axonal growth was quantified in comparison with controls. Expression of 13 genes (4.2%) promoted significantly increased axonal growth with no obvious adverse effects on neuronal survival or axonal trajectory (Figures 2A–E). In a second selection step (Figure 1B), these 13 genes were evaluated in an acute sLNv axonal injury model in *Drosophila* brains explanted and kept in culture (Ayaz et al., 2008; Koch, 2012). Given their superficial location, sLNv axons are easily accessible for injury, and were physically severed using an ultrasonic microchisel. Using the temperature dependency of the UAS/Gal4 system, high expression levels of candidate genes were induced in adult flies starting at 24 h before injury. Axonal regrowth was defined as the growth of novel sprouts from the site of injury within 4 days. We used three parameters to evaluate axonal regrowth following injury: capacity of regrowth (the percentage of brains that exhibited at least one axonal sprout grown de novo), total regrowth (defined as the sum of the lengths of novel sprouts), and the maximum projection distance (defined as the distance of the longest novel sprout from the site of injury to their terminus) (Figures 3A–D). Of the genes tested, seven (*drl, Dscam1, faf*, *kay, pdm2, pum*, and *sens*) showed enhanced regeneration in all three categories (Figures 3A–H). Kayak is the fly homolog of Fos, a key transcription factor downstream of JNK signaling, confirming that the screen can identify bona fide regeneration genes. Dichaete, D, is an example of a gene that promoted axonal outgrowth during development (Figures 2A,D), but failed to induce regeneration in most cases (Figures 3A,G) and often resulted in short sprouts with poor morphology, making it difficult to measure. Three genes (*dimm*, d*ac*, and s*qz*) caused axonal phenotypes such as defasciculation, blebbing, or fragmentation, and were excluded from further analysis.

### The ability of faf to induce axonal regrowth is conserved and depends on its enzymatic activity

**Figure 4:**
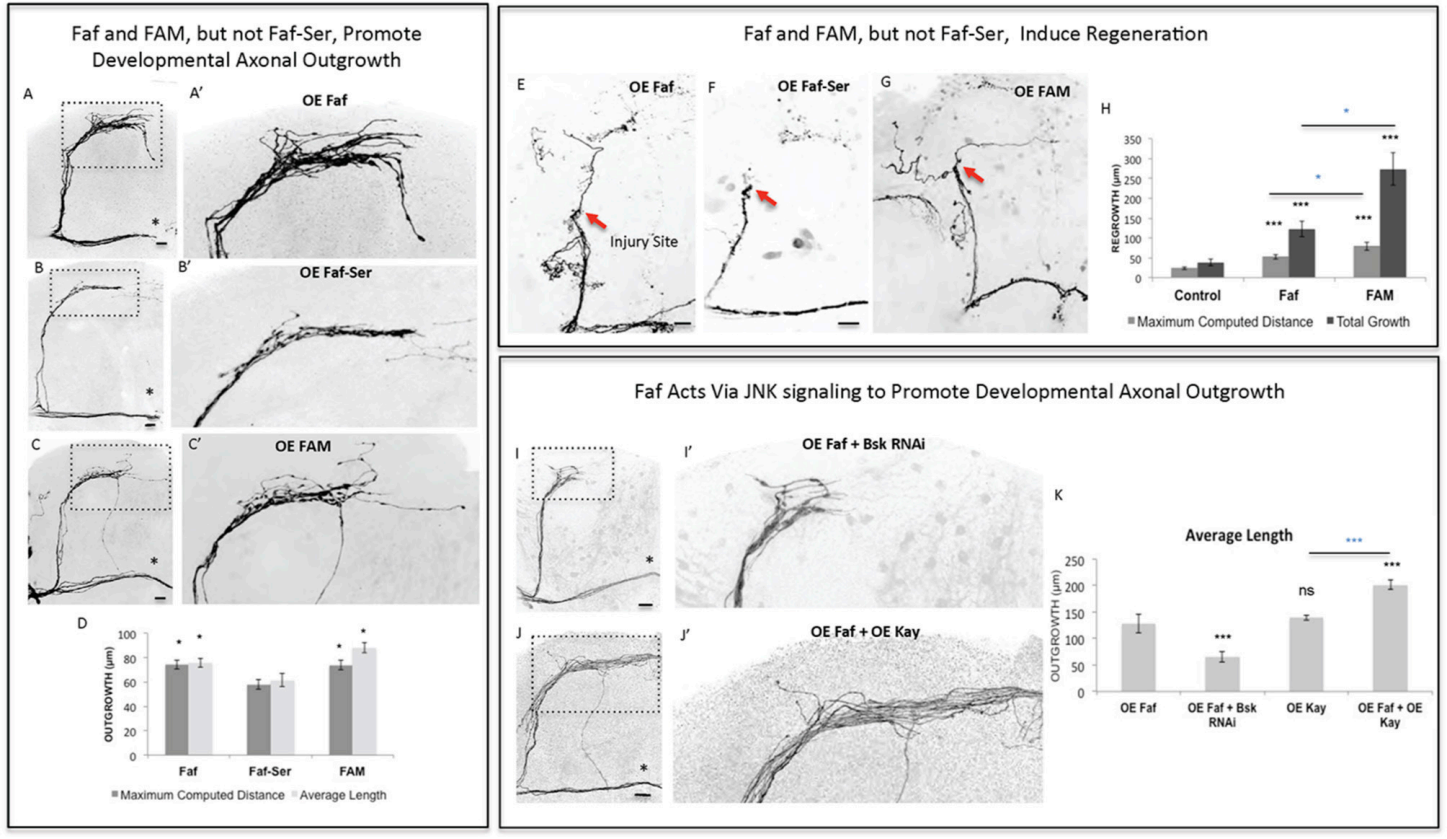
Faf and FAM, but not Faf-Ser, promote axonal outgrowth in development and axonal regrowth after injury, and interact with the JNK signaling pathway. **(A–C)** Representative images of sLNv axonal arborization in adult flies where developmental overexpression of *Fat facets, faf* **(A,A')**, *Fat-Serine, Faf-Ser* **(B,B')**, and *FAM* **(C,C')** has been specifically induced in the sLNvs. **(D)** Morphometric analysis (Maximum Computed Distance and Average Length) of sLNv axonal projections for **(A–C)**. Axonal outgrowth is measured in μm. **(E–G)** Representative images of sLNv axonal regrowth 4 days after injury in flies where overexpression of *faf* **(E)**, *Faf-Ser* **(F)**, and *FAM* **(G)** has been specifically induced in the sLNvs. **(H)** Morphometric analysis (Maximum Computed Distance and Total growth) of regenerated sLNv axonal projections in **(E–G)**. Axonal outgrowth is measured in μm. **(I,J)** Representative images of sLNv axonal arborization for epistasis experiments between *faf* and *Bsk* **(I,I')** and *faf* and *kay* **(J,J')**. **(K)** Morphometric analysis (Average Length) of sLNv axonal projections where developmental overexpression of *faf*; *faf* and *Bsk* RNAi; *kay*; and *faf* and *kay*, has been specifically induced in the sLNvs. Axonal outgrowth is measured in μm. Genotype of flies in **(A,A',E)** is PDF-Gal4, UAS-GFP/+; PDF-Gal4, UAS-2x eGFP/+; UAS-Faf/+, in **(B,B',F)** is PDF-Gal4, UAS-GFP/+; PDF-Gal4, UAS-2x eGFP/+; UAS-Faf-Ser/+, in **(C,C',G)** is PDF-Gal4, UAS-GFP/+; PDF-Gal4, UAS-2x eGFP/+; UAS-FAM/+, in **(I,I')** is PDF-Gal4, UAS-GFP/+; PDF-Gal4, UAS-2x eGFP/+; UAS-Faf/UAS-Bsk RNAi, in **(J,J')** is PDF-Gal4, UAS-GFP/+; PDF-Gal4, UAS-2x eGFP/+; EP-Faf/UAS-kay. Dotted insets have been zoomed in to better illustrate the diverse axonal phenotypes obtained. Asterisk denotes the brain midline, red arrow denotes the injury point. ^*^p < 0.05; ^***^p < 0.001. n.s. indicates no statistical significance. Error bars represent SEM. Dotted insets have been zoomed in to better illustrate the diverse axonal phenotypes obtained. OE indicates overexpression. Scale bars are 20 μm.

Of the identified seven genes, three in particular (*Dscam1, faf* and *kay*) appeared to consistently promote the most growth across all criteria. We therefore asked whether these genes might be acting together in a novel regeneration pathway linking the cell surface to the nucleus. We began by analyzing the de-ubiquitinating enzyme Faf since it promoted the highest levels of regeneration across all criteria (Figures 3A–C,H). First, we confirmed that *faf* is also able to induce axonal overgrowth in other CNS neuronal populations, such as the Dorsal Cluster Neurons (DCNs) (Figure 3—Supplementary Figure 1), confirming that Faf may be a general CNS axonal growth-promoting factor. Ubiquitin-dependent protein regulation is critical in regulating many neuronal events, including axonal growth (McCabe et al., 2004; Ambrozkiewicz and Kawabe, 2015). However, the signaling pathways operating downstream of these enzymes are still largely unknown. To test whether the axonal growth induced by Faf was dependent on its deubiquitinase activity, we mutated a critical cysteine 1,677 residue in the catalytic protease site to a serine (Chen and Fischer, 2000). In contrast to wild-type Faf, this mutated form of Faf was not able to significantly promote developmental axonal growth (Figures 4A,B,D). The mouse homolog of Faf, FAM/Usp9x, which can be active in *Drosophila* in other contexts (Wood et al., 1997; Chen et al., 2000), also induced robust sLNv axonal outgrowth (Figures 4C,D). Remarkably, even the yeast homolog of Faf, Ubp2, which only shares homology in the de-ubiquitination domain, induces sLNv axonal outgrowth very similar to Faf (Figure 4—Supplementary Figure 2). More importantly, both FAM and Faf, but not the enzymatic mutant Faf-Ser, induced significant axonal regeneration after injury (Figures 4E–H). These data suggest a conserved axonal growth and regeneration activity for Faf as a deubiquitinase enzyme.

### Faf promotes axon regrowth in a JNK-dependent manner

Faf has been shown to induce neuromuscular junction growth in *Drosophila* (DiAntonio et al., 2001) in a pathway that requires Wallenda (Wnd), a conserved MAPKK upstream of JNK signaling (Collins et al., 2006). Therefore, we tested whether Faf required Wnd to induce axonal growth. RNA interference knock-down (RNAi KD) of *wnd* inhibited Faf-mediated axonal outgrowth (Figure 4—Supplementary Figures 3A,B,I), whereas overexpression of *wnd*, but not a kinase-dead form of it, strongly promoted axonal outgrowth that essentially phenocopied *faf* overexpression (Figure 4—Supplementary Figures 3A,C,D). Moreover, overexpression of *wnd* also promoted axonal regrowth after injury (Figure 4—Supplementary Figures 3G,H,J). Therefore, Wnd likely acts downstream of Faf, to modulate axonal growth and regeneration in response to *faf* overexpression. Similarly, RNAi KD of the *Drosophila* homolog of JNK, *basket* (*bsk*), completely inhibited *faf*-mediated axonal outgrowth (Figures 4I,K). Conversely, co-expression of *kay*, the JNK pathway effector we identified as strong promoter of outgrowth in development (Figures 2A,C) and after injury (Figures 3A–C,F) enhanced Faf-mediated axonal outgrowth (Figures 4J,K). Together, these data suggest that Wnd and JNK act downstream of Faf to induce axonal outgrowth and regeneration.

### Faf stabilizes Dscam1 protein levels to promote axonal growth

How might *faf* activate JNK signaling to induce axonal regeneration? During fly eye development *faf* mediates the internalization of the Notch ligand Delta (Overstreet et al., 2004), and Notch signaling has been proposed to enhance regeneration of developing neurons (Kato et al., 2011), though it has also been shown to act as a repressor of axonal regeneration (El Bejjani and Hammarlund, 2012). To test if *faf* interacted with Delta in the context of sLNv axonal growth, we tested both a RNAi KD as well as a dominant negative (DN) transgene, and found that loss of Delta function in the sLNvs did not reduce the axonal outgrowth activity of *faf* (data not shown), suggesting an alternative mechanism in the context of axonal growth. Therefore, we reasoned that Faf might interact with different axon growth-promoting effectors.

**Figure 5:**
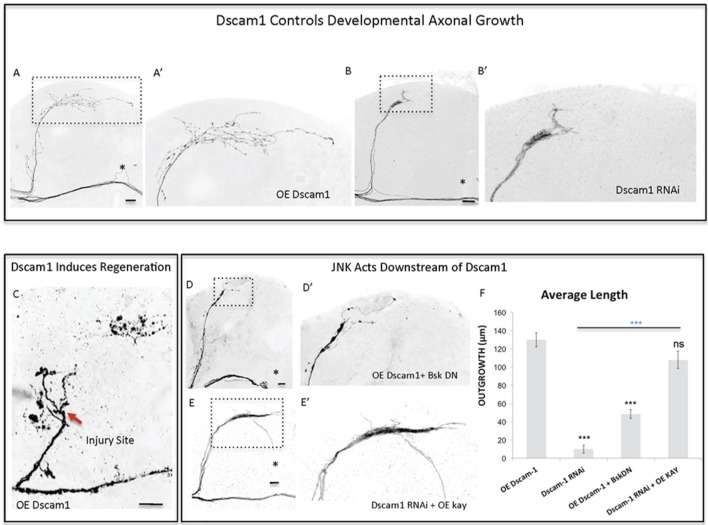
Dscam1 promotes axonal outgrowth in development and axonal regrowth after injury, and interacts with the JNK signaling pathway. **(A,B)** Representative images of sLNv axonal arborization in adult flies where developmental overexpression of *Dscam1* **(A,A')**, and Dscam1 RNAi **(B,B')** has been specifically induced in the sLNvs. **(C)** Representative image of sLNv axonal regrowth 4 days after injury in flies where overexpression of *Dscam1* has been specifically induced in the sLNvs. **(D,E)** Representative images of sLNv axonal arborization demonstrating that inhibition of *Bsk* accomplished by overexpression of a dominant negative line of *Bsk* inhibits Dscam1- induced outgrowth **(D,D')**, and that overexpression of *kay* rescues the lack of axonal growth induced by overexpression of Dscam1-RNAi **(E,E')**. **(F)** Morphometric analysis (Average Length) of sLNv axonal projections where developmental overexpression of *Dscam1*; Dscam1-RNAi; *Dscam1* and Bsk DN; Dscam1-RNAi and *kay*, has been specifically induced in the sLNvs. Axonal outgrowth is measured in μm. Genotype of flies in **(A,A',C)** is PDF-Gal4, UAS-GFP/+; PDF-Gal4, UAS-2x eGFP/+; UAS-Dscam1-HA/+, in **(B,B')** is PDF-Gal4, UAS-GFP/+; PDF-Gal4, UAS-2x eGFP/+; UAS-Dscam1-RNAi/+, in **(D,D')** is PDF-Gal4, UAS-GFP/+; PDF-Gal4, UAS-2x eGFP/UAS-Bsk-DN; UAS-Dscam1-HA/+, in **(E,E')** is PDF-Gal4, UAS-GFP/+; PDF-Gal4, UAS-2x eGFP/+; UAS-Dscam1-RNAi /UAS-kay. Dotted insets have been zoomed in to better illustrate the diverse axonal phenotypes obtained. Asterisk denotes the brain midline, red arrow denotes the injury point. ^*^p < 0.05; ^***^p < 0.001. n.s. indicates no statistical significance. Error bars represent SEM. Dotted insets have been zoomed in to better illustrate the diverse axonal phenotypes obtained. OE indicates overexpression. Scale bars are 20 μm, with exception of C, which is 30 μm.

Mammalian *Dscam1* (Qu et al., 2013) has been shown to be a regulator of JNK signaling. Interestingly, our screen identified *Dscam1* as one of the genes that most strongly and consistently promoted sLNv axonal outgrowth and regeneration (Figures 2A, 3A–D). This prompted us to investigate the molecular mechanisms underlying the growth and regeneration activity of *Dscam1*. The *Dscam1* gene generates a large number of isoforms by alternative splicing of a plethora of extracellular domains and two transmembrane domains called TM1 and TM2 (Schmucker et al., 2000). We find that different isoforms containing either the TM1 (Figures 2A, 5A), or the TM2 domain (not shown), and different extracellular domains (UAS-Dscam1 1.30.30.1-GFP and UAS-Dscam1 1.34.31.1-HA, see Methods) can induce axonal outgrowth, suggesting that induction of axonal growth may be a general property of Dscam1-mediated signaling independent of its isoform specificity. It has previously been reported that isoforms containing TM1 are dendrite specific (Shi et al., 2007). However, we find that upon overexpression these isoforms localize to both cell bodies and axonal terminals (Figure 5—Supplementary Figure 4). Conversely, *Dscam1* knock-down with two different RNAi lines (Watson et al., 2005) resulted in stunted sLNv axonal growth (Figures 5B,F). Finally, TM1-containing Dscam1 isoforms, UAS-Dscam1 1.30.30.1-GFP and UAS-Dscam1 1.34.31.1-HA induce robust axonal regeneration after injury, with the latter being the strongest line (Figure 5C).

Both mammalian and fly Dscam1 are known to interact with p21 activating kinase (Pak) (Schmucker et al., 2000) itself an upstream JNK Kinase. We find that inhibition of JNK activity, by using a dominant negative form of Bsk, completely abrogates Dscam1 mediated axonal growth (Figures 5D,F). Conversely, the expression of Kay reverses the loss of axon growth caused by *Dscam1* RNAi knock-down (Figures 5E,F). These data suggest that Dscam1 acts upstream of JNK signaling to induce axonal growth.

**Figure 6:**
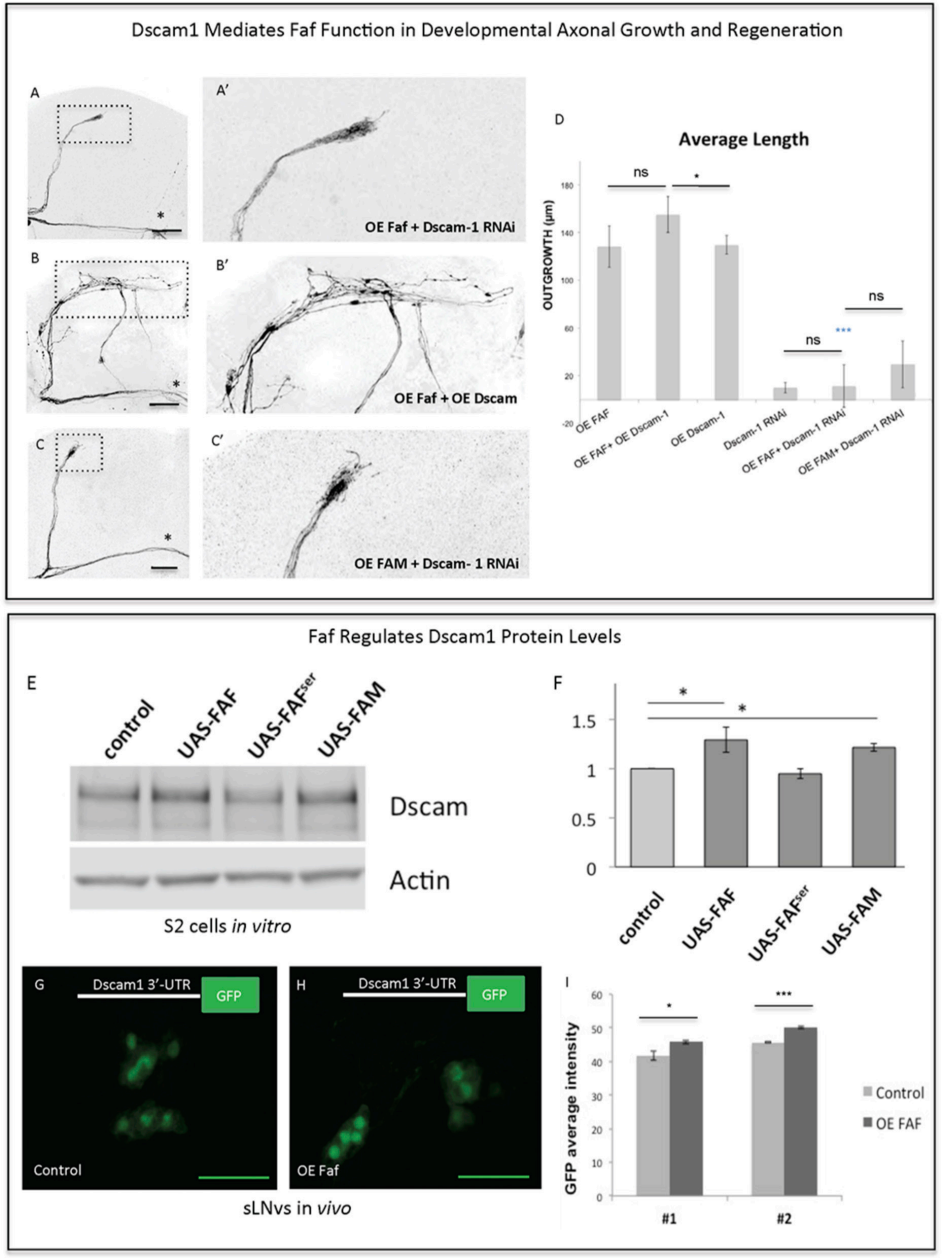
Faf and Dscam genetically and biochemically interact. **(A–C)** Representative images of sLNv axonal arborization demonstrating that knock-down of *Dscam1* inhibits Faf- induced outgrowth **(A,A')**, that co-overexpression of both *faf* and *kay* potentiates axonal growth **(B,B')** and that knock-down of *Dscam1* inhibits FAM-induced outgrowth. **(D)** Morphometric analysis (Average Length) of sLNv axonal projections where developmental overexpression of *faf*, *FAM, Dscam1* and Dscam1 RNAi has been specifically induced in the sLNvs, uncovering gene interactions. Axonal outgrowth is measured in μm. **(E,F)** Western blot and quantification showing increased levels of Dscam1 protein following S2 electroporation of wild-type Faf and FAM, but not of Faf-Ser in comparison to control (UAS vector). **(G–I)** GFP fluorescence analysis showing increased levels of GFP from a GFP-3′UTR-Dscam1 construct following overexpression of *faf* (*n* = 28) **(H)** in comparison to its control (*n* = 24) **(G)**. LNVs GFP average intensities are shown in **(I)**. Genotype of flies in **(A,A')** is PDF-Gal4, UAS-GFP/+; PDF-Gal4, UAS-2x eGFP/+; / UAS-Faf/UAS-Dscam1-RNAi, in **(B,B')** is PDF-Gal4, UAS-GFP/+; PDF-Gal4, UAS-2x eGFP/+; / UAS-Faf/UAS-Dscam1-HA, in **(C,C')** is PDF-Gal4, UAS-GFP/+; PDF-Gal4, UAS-2x eGFP/+; UAS-FAM/ UAS-Dscam1-RNAi, in **(G)** is PDF-Gal4, UAS-GFP/+; UAS-3′-UTR-Dscam1-GFP/+; UAS-Faf/+, in **(H)** is PDF-Gal4, UAS-GFP/+; UAS-3′-UTR-Dscam1-GFP/+; TM6b/+. Dotted insets have been zoomed in to better illustrate the diverse axonal phenotypes obtained. Asterisk denotes the brain midline, ^*^p < 0.05; ^***^p < 0.001. n.s. indicates no statistical significance in **(D)**. Error bars represent SEM in **(D)** and in **(I)**. OE indicates overexpression. Scale bars in **(A–C)** and **(G–H)** are 30 μm.

The fact that Faf and Dscam1 both promote axonal regeneration after injury and induce strikingly similar JNK-dependent axonal outgrowth phenotypes led us to hypothesize that Faf and Dscam1 interact in this context. Indeed, we find that Faf-induced axonal outgrowth required Dscam1, as *Dscam1* knock-down almost completely abolished Faf-induced growth (Figures 6A,D). Consistent with this, co-overexpression of *faf* and *Dscam1* in the sLNvs induces stronger axonal outgrowth than *faf* overexpression alone (Figures 6B,D). Importantly, *Dscam1* knock-down also inhibits FAM/Usp9x mediated axonal outgrowth, indicating a conserved interaction (Figures 6C,D).

Faf antagonizes ubiquitination by cleaving the covalent bond between ubiquitin and a substrate protein (Huang et al., 1995), thereby leading to stabilization of proteins targeted for degradation. We asked if Faf might stabilize Dscam1 protein levels. Therefore, we expressed *Dscam1* alone or together with *faf, faf-Ser* mutant or mouse *FAM/Usp9x* in *Drosophila* S2 cells. Both Faf and FAM/Usp9x, but not the Faf-Ser mutant lead to a ~30% increase in Dscam1 protein levels (Figures 6E,F), with no change in mRNA levels. However, we were unable to find evidence for Dscam1 ubiquitination in wild type or proteasome-inhibited S2 cells, nor a change in that status upon overexpression or knock-down of *faf* (data not shown). These data suggest that, at least in this context, Faf does not de-ubiquitinate Dscam1 directly.

Dscam1 has also been shown to be post-transcriptionally regulated via the *Drosophila* DLK1 homolog Wnd, in particular, through stabilization of its 3′-UTR. Specifically, using a *Dscam1-3*′*-UTR*>*GFP* reporter construct it has been shown that translation of the reporter protein is enhanced by Wnd (Kim et al., 2013). Since our data so far indicate that Faf acts upstream of both Dscam1 and Wnd (Figure 4—Supplementary Figure 2), we tested whether Dscam1 is also required for Wnd-induced axonal growth in sLNv neurons. We find that Dscam1 RNAi KD significantly decreased Wnd-induced growth (Figure 4—Supplementary Figures 3E,F). We then asked whether Faf enhances translation of Dscam1 through a mechanism that is mediated by the 3′UTR of *Dscam1 in vivo*. To this end, we expressed the *Dscam1-3*′*-UTR*>*GFP* reporter in the sLNv alone or together with *faf*. We found that GFP levels are significantly upregulated in sLNv upon Faf overexpression (Figures 6G–I).

## Discussion

**Figure 7:**
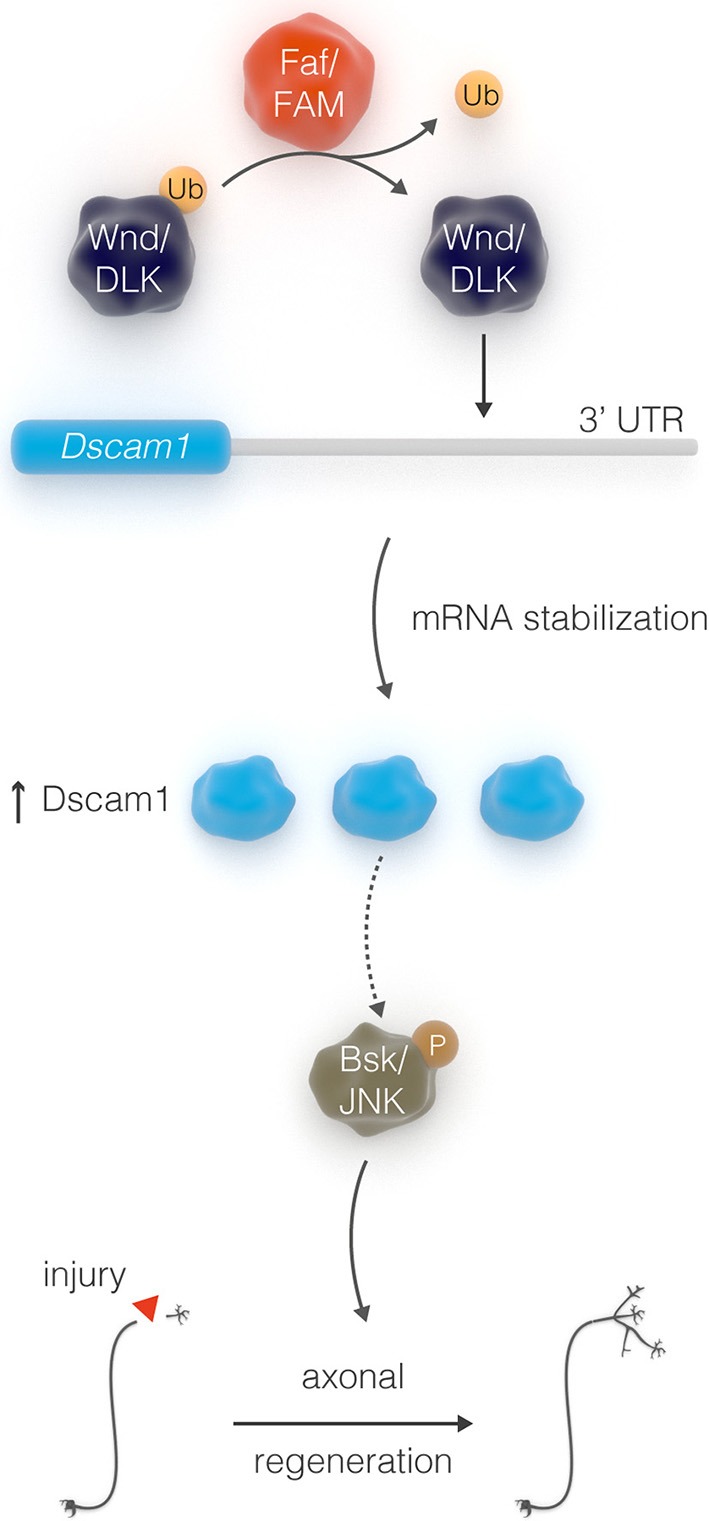
Schematic depicting the interactions between Faf, Dscam, and JNK in axonal outgrowth.

In contrast to young neurons, injured adult CNS neurons exhibit very limited ability to self-repair, suggesting that the intrinsic regenerative capacity is lost during development. For example, it has been shown that the axon growth rate decreases dramatically with age in post-natal retinal ganglion cells (Goldberg et al., 2002). In addition, pioneer work from Filbin and colleagues demonstrated that developmental loss of the regenerative capacity of neurons in post-natal rats is mediated by a decline in the endogenous levels of neuronal cAMP within a few days after birth (Cai et al., 2001). Consistent with this evidence, transcription factors that regulate developmental axonal growth, such as members of the Kruppel-like family (KLFs), can promote regrowth of adult injured corticospinal tract and optic nerve axons (Moore et al., 2009; Blackmore et al., 2012). Several other intrinsic axonal regulators, including phosphatase and tensin homolog (PTEN), suppressor of cytokine signaling 3 (SOCS3), mTOR, Osteopontin, and IGF-1 (Liu et al., 2010; Sun et al., 2011; Duan et al., 2015), have been previously identified in mammalian systems—though mostly on a gene by gene basis. More systematic approaches, such as quantitative proteomic analysis, have been recently employed to identify molecular pathways that are altered in injured retinal ganglion cells, and identified additional intrinsic regulators of regeneration, such as c-Myc (Belin et al., 2015). Taken together, these studies suggest that manipulation of the intrinsic regenerative ability of mature neurons might be an efficient strategy for enhancing the capacity of injured axons to regenerate. It is therefore crucial to discover factors that constitute intrinsic pro-regeneration signaling pathways in a systematic manner. We have previously shown that the adult *Drosophila* CNS is a suitable model for studying axonal injury and regeneration (Ayaz et al., 2008). By exploiting this model along with the power of *Drosophila* genetic screens, we have uncovered a novel axonal regeneration pathway that links the stability of the neuronal cell surface receptor Dscam1, via the de-ubiquitination function of the enzyme Faf, to JNK signal (Figure 7), a major inducer of axonal regeneration in *C. elegans, Drosophila*, and mouse (Raivich et al., 2004; Raivich and Makwana, 2007; Ayaz et al., 2008; Nix et al., 2011; Li et al., 2012).

E3 ubiquitin ligases are important in many aspects of mammalian brain development and function, by controlling neuritogenesis, modulating axon guidance and pruning, neuronal polarity and synaptic transmission (Ambrozkiewicz and Kawabe, 2015). Not surprisingly, E3 ligase dysfunction and abnormal ubiquitin signaling is implicated in several human brain disorders. In particular, mutations in the mammalian homolog of Faf, FAM/Usp9x, have been associated with X-linked intellectual disability (Homan et al., 2014). Brain specific deletion of FAM/Usp9x results in early postnatal death, and FAM/Usp9x knock-out neurons display reduced axon growth and impaired neuronal migration (Stegeman et al., 2013; Homan et al., 2014).

Ubiquitin-dependent signals that include Faf have also been shown to regulate synaptic development and growth at the *Drosophila* neuromuscular junction (NMJ) (DiAntonio et al., 2001), though its function in the CNS remained elusive. Both yeast, Ubp2, and mouse homologs of Faf display the ability to induce axonal growth in the CNS, suggesting conservation of this property throughout evolution, similar to what had been shown at the NMJ (DiAntonio et al., 2001; Kim et al., 2013). Importantly, we show for the first time that both Faf and FAM promote regrowth of injured axons in the adult fly brain.

Ubiquitin signaling is rather complex, and dissecting it downstream players can be challenging. In our screen, overexpression of the neuronal cell adhesion Dscam1 induced robust axonal growth both in development as well as after injury similar to the one induced by Faf, which led us to hypothesize that both genes acted in the same growth-promoting signaling pathway. In *Drosophila*, Dscam1 shows extensive molecular diversity that results from alternative splicing into some 18500 diverse extracellular domains (Schmucker et al., 2000). This isoform diversity has been shown to be critical for neuronal self-recognition and self-avoidance underlying axon growth and dendritic patterning (Hughes et al., 2007; He et al., 2014). Independent of its ectodomain diversity, Dscam1 has also been recently shown to regulate presynaptic arbor growth (Kim et al., 2013).

Our data suggest that post-transcriptional regulation of Dscam1 allows axonal growth after injury. The Dscam1-stabilizing function of Faf appears to be conserved in mammals, as FAM/Usp9x overexpression also leads to increased levels of Dscam1 protein DLK1/Wnd levels and activity are known to be regulated by the ubiquitin ligase *Highwire* in Drosophila (Wu et al., 2007). Interestingly, Wnd itself is a promoter of mRNA stability and local translation, and is essential for axon regeneration after laser axotomy in adult neurons in *C. elegans* (Yan et al., 2009; Byrne et al., 2014). Although it remains unclear how, at the molecular level, DLK1 recognizes and regulates the 3′-UTR of Dscam1, the placing of Faf upstream of Wnd and Dscam1 in regulating axonal outgrowth, together with the fact that the de-ubiquitinating activity of Faf is likely required for this function, suggests that Faf may operate by antagonizing the E3 ubiquitin ligase activity of enzymes such as Highwire (DiAntonio et al., 2001).

## Methods

### Candidate gene sample

We used the Gene Ontology tool (http://geneontology.org/) to select candidate genes annotated with the terms “Neurite Morphogenesis,” “Transcription Factors,” “Receptors,” “Chromatin Modifiers,” and “Ubiquitin Ligases.” We also included an additional set of genes previously implicated in axonal growth and/or involved in actin dynamics (indicated in Supplementary Table 1 by asterisks).

The final candidate gene sample only included genes for which appropriate gain- of-function fly lines were readily available at the stock centers at the time of the study (Supplementary Table 1).

### Fly stocks and genetics

*Drosophila melanogaster* stocks were kept on standard cornmeal media. For tissue-specific overexpression of the transgenes, we used the GAL4/UAS system (Brand and Perrimon, 1993). Lines with UAS insertion sites (i.e., UAS, EP, EPgy2, XP, and Mae-UAS) were received through the Bloomington or Szeged Stock Centres (or from specific laboratories when specified). Loss-of-function lines [Wnd RNAi GD8365, Dscam1 RNAi KK108835; Dscam1 RNAi (Watson et al., 2005); Bsk RNAi BL35594 and BL36643, and Bsk DN BL6409] were obtained from the Bloomington Stock Centre or from the Vienna *Drosophila* Research Centre (VDRC). UAS-Wnd kinase dead (KD) and UAS-Wnd E flies were a gift from C. Collins. The *PDF-Gal4* line was obtained from P. Taghert. For Faf overexpression in flies, we used the EP3520 line (Szeged Stock Centrum), which was previously reported to induce Faf gain-of-function (DiAntonio et al., 2001). UAS-Faf and UAS-FAM lines were created in house by cloning Faf cDNA and FAM cDNA into a pUAST-attB vector, respectively (Bischof et al., 2007) and injected in an attP2 docking line (BL 8622). A UAS-Faf serine (Faf-Ser) mutant that harbors a cysteine to serine mutation at residue 1677 was also cloned using the same method. UAS-Dscam1 HA-FLAG and UAS-Dscam1 GFP flies, both containing the 1.30.30.1 isoform, were used for the injury experiments. The UAS-Dscam1 1.30.30.1 GFP flies have been described (Hughes et al., 2007) and the UAS-Dscam1 HA-FLAG flies were created by inserting a HA tag into the intracellular domain (after the 81st bp of exon 22) of isoform 1.34.31.1. The UAS-Dscam1 1.30.30.1 GFP flies have been used for the initial screen in development and after injury, and the UAS-Dscam1 1.30.30.1 HA has been used for additional confirmatory experiments in development and injury, as well as for epistasis experiments. Although both lines induced significant axonal growth in development and after injury, UAS-Dscam1 1.30.30.1 HA appeared to be the strongest of the two lines. For the genetic screen in development and after injury, pdf-Gal4, UAS-GFP; pdf-Gal4, UAS-2x eGFP/cyo flies were kept as a stock and used to drive expression of the various candidate genes, or crossed to wild-type Canton S (CS) flies. For the genetic epistasis experiments, pdf-Gal4, UAS-GFP; UAS-Dscam1 RNAi and pdf-Gal4, UAS-GFP; Faf (EP 3520) flies were maintained as a stock and crossed to overexpression lines to uncover genetic interactions.

### Developmental outgrowth screen

To measure axonal outgrowth during development, flies were reared at 25°C and were dissected 2–10 days after eclosion. A minimum of five fly brain (10 sLNs projections) per genotype were stained with an anti-GFP antibody (to enhance the GFP signal), visualized under a fluorescent microscope equipped with a GFP filter and scored as “growth” (when sLNv axonal projections appeared considerably longer than in controls) or “no-growth” (when the length of sLNv projections was indistinguishable from controls or shorter). All genes that were scored growth promoting genes were confirmed as such in at least one independent experiment, and their growth inducing ability was analyzed by measuring the axonal sLNv dorsal axonal projections.

### Whole brain explant culture injury system

For the axonal regrowth analysis after injury, flies were reared at 18°C, in order to minimize overexpression effects during development, and shifted to 25°C the day before injury to allow optimal transgene expression.

Whole-brain explants on culture plate inserts were prepared and injured as described (Ayaz et al., 2008; Koch, 2012). In brief, Millicell low height culture plate inserts (Milipore) were coated with laminin and poly-lysine (BD Biosciences). Adult female flies were collected 2–10 days after eclosion and placed on ice. Fly brains were quickly and carefully dissected out in a sterile Petri dish containing ice cold Schneider's *Drosophila* Medium (GIBCO). Up to seven brains were placed on the membrane of one culture plate insert and culture medium (10,000 U/ml penicillin, 10 mg/ml streptomycin, 10% Foetal Bovine Serum, and 10 μg/ml insulin in Schneider's *Drosophila* Medium) was added. sLNv axonal injury was performed using an ultrasonic microchisel controlled by a powered device (Eppendorf). Culture dishes were kept in a plastic box in a humidified incubator at 25°C.

### Immunohistochemistry

Freshly dissected brains of adult flies were fixed in 4% formaldehyde and processed for immunohistochemistry as described (Hassan et al., 2000). Cultured brains (4 days post-injury) were first fixed by replacing the culture medium in the Petri dish for 30 min. Then, 1 ml of fixative was carefully added on top of the filter for 1–2 h. Brains that detached from the membrane were excluded from further analysis. Immunostaining was performed as for freshly dissected samples. Primary antibodies were rabbit anti-GFP (A-6455, Molecular Probes), rat anti-HA (3F10, Roche); and anti-Pdh (gift from P. Taghert).

### Imaging and morphological analysis of sLNv axonal projections in development and after injury

Image J software was used to measure the length of the dorsal axonal projections emanating from the sLNvs. The starting point was set as the point where axons turn medially and start to run parallel to the commissure. Axonal length was measured as a straight line (Computed Distance) from the starting point toward the midline (indicated by an asterisk) and as manual trace using Image J. The maximum computed distance was defined as the distance projected by the longest axonal sprout in a straight line and parallel to the commissure. The Average Length was the defined as the average length of the two longest axonal branches traced manually (freehand distance). Imaging was performed on an upright Zeiss Axioscope equipped with a CCD camera, or on a Zeiss 700 or Nikon AR1 confocal microscope. All measurements were performed using ImageJ.

To analyze the role of the candidate genes in axonal regrowth after injury we imaged cultured brains at two different time points after injury: approximately 5 h and 4 days. Comparison between these two timepoints allowed us to define the location at which the injury took place, in order to define *de novo* growth. Morphometric analysis of axonal regrowth was always performed 4 days after injury, following fixation and GFP staining of the brains in culture. Capacity of regrowth was defined as the ability of the injured sLNv projection to regrow at least one new axonal sprout. Without the support of the head cuticle, brains will flatten and therefore undergo slight morphological changes during the culture process. To be conservative and account for potential inaccuracies in defining the injury point, only regrown axons with a minimum length of 12 μm were defined as de novo growth and taken into account for analysis. To quantify axonal regrowth, newly grown axons were measured in a straight line and manually traced using ImageJ. In this case, the maximum computed distance was defined as the average of the distance of the two longest axonal sprouts in a straight line in any direction. Maximum growth was defined as the sum of the freehand lengths of all de novo grown axons. Images 5 h after injury were acquired on an upright Nikon microscope equipped with a Hamamatsu CCD camera ORCA-R2. Imaging 4 days after injury was performed on a Zeiss 700 or a Nikon A1R confocal microscope after GFP immunostaining. See figure legends for details of individual experiments, including statistical tests used and see Supplementary Table 2 for the number of samples tested.

### Cell culture and western blotting

Drosophila Schneider's (S2) cells were maintained in Sf-900 II SFM medium (Gibco). To achieve transgene overexpression in Schneider's (S2) cells we electroporated a UAS construct in combination with PMT-Gal4, according to previously developed methods (Klueg et al., 2002). For Faf and FAM overexpression, we created a UAS-Faf and a UAS-FAM construct as described in “Fly stocks and Genetics.” For Dscam1 overexpression, the UAS-Dscam1 1.30.30.1 GFP construct was used (Hughes et al., 2007).

Cells were electroporated using an Amaxa Nucleofector KitV (Lonza), according to the manufacturer's instructions. Cells were harvested 72–96 h after copper induction, briefly washed with PBS and pellets frozen until cells were lysed in a 1% NP40 buffer in Tris-HCL. Protein concentration was determined by a modified Lowry assay (Peterson, 1977). Western blotting was performed with a SDS-PAGE Electrophoresis System (Biorad). Briefly, protein samples were diluted in SDS containing sample buffer and 15 μg per sample was loaded onto a 3–8% Tris-Acetate mini gel (Novex, Life Technologies). Samples were blotted using tank transfer to a nitrocellulose membrane (GE Healthcare), blocked with milk and probed with primary antibodies against Dscam1 (1:1,000) (Watson et al., 2005) or against actin (1:5,000, ab3280, Abcam), which was used as a protein loading control. Anti-rabbit or anti-mouse horseradish peroxidase conjugated secondary antibodies (Amersham) were then added, and proteins were detected using enhanced chemiluminescence (ECL Plus, GE Healthcare) on a FUJI LAS imager system (Fuji). Values for Dscam1 were normalized to the values of the loading control (actin) and quantified using the blot analysis function for IMAGE J. Kruskall Wallis test was used to compare the different conditions. Data is shown as mean ± SEM and significance was set at *p* ≤ 0.05.

### RNA isolation and quantitative PCR

RNA was extracted with Trizol. 1 μg of total RNA was reverse transcribed using the Quantitect RT kit (Qiagen). qPCR was performed using the Taqman Real Time protocol (Applied Biosystems) and probes. Data is shown as mean ± SEM.

### GFP intensity measurements

Adult brains were dissected and immediately prepared for imaging. Confocal stacks of all sLNv and lLNv cell bodies in each side of the brain were performed. The optimal confocal settings were first adjusted for wild type brains and kept unchanged to allow comparison between genotypes. A maximum projection was created for each brain side and each image was quantified for GFP intensity using the “Image Analysis” module of Zeiss Zen 2.0 software. All quantifications were done by an investigator blind to experimental conditions. Student *t*-test was used to compare both genotypes. Data is shown as mean ± SEM and significance was set at *p* ≤ 0.5.

## Author contributions

MK and BH designed the research. MK, MN, and BH analyzed the data. MH contributed expertise and reagents. MK, DS, and BH wrote the manuscript. MK, MN, MZ, NdG, AC, JY, M-LE, and MM performed experiments.

### Conflict of interest statement

The authors declare that the research was conducted in the absence of any commercial or financial relationships that could be construed as a potential conflict of interest.

## Supplementary material

Supplementary Figure 1 Faf gain of function promotes axonal growth in a distinct neuronal population. **(A,B)** Overexpression of *faf* specifically in the Dorsal Cluster Neurons (DCNs) results in increased axonal growth (yellow arrows) **(B)**, in comparison to wild-type flies **(A)**. Genotype of flies in **(A,A**′**)** is;UAS-GFP;ato-Gal4 14a, in (**B,B**′);UAS-GFP;ato-Gal4 14a/UAS-Faf. Scale bars are 20 μm.

Supplementary Figure 2 Ubp2 gain of function also promotes axonal growth. **(A,B)** Overexpression of the yeast homolog of Faf, *Ubp2*, which shows conservation of the enzymatic domain, also results in increased axonal growth **(B)**, in a similar manner to Faf overexpression **(A)**. Note that **(B,B**′**)** are the same as in Figures 4A,A'. Genotype of flies in (**A,A'**) is PDF-Gal4, UAS-GFP/+; PDF-Gal4, UAS-2x eGFP/+; UAS-Faf/+; in (**(B,B**′) is PDF-Gal4, UAS-GFP/+; PDF-Gal4, UAS-2x eGFP/UAS-Ubp2. Dotted insets have been zoomed in to better illustrate the diverse axonal phenotypes obtained. Scale bars are 30 μm.

Supplementary Figure 3 Wallenda promotes growth in development and after injury and is required for Faf-induced growth. **(A,B)** Representative images of sLNv axonal arborization demonstrating that knock-down of *wnd* inhibits Faf-induced outgrowth. **(C,D)** Representative images of sLNv axonal arborization in adult flies where developmental overexpression of *wnd* **(C,C**′**)**, but not of a kinase dead form **(D,D**′**)** in the sLNvs induces axonal growth similar to the one induced by Faf **(A,A**′**)**. **(E,F)** Representative images of sLNv axonal arborization demonstrating that knock-down of *Dscam1* inhibits Wnd-induced outgrowth **(E,E**′**)** and results in a phenotype that resembles knock-down of *Dscam1* on its own **(F,F**′**)**. **(G,H)** Overexpression of *wnd* in the sLNVs **(G,G**′**)**, but not of Wnd KD **(H,H**′**)** induces axonal regrowth 4 days after injury. Five hour after injury timepoints **(G,H)** have been included to better illustrate the regenerative ability of Wnd, but not of its kinase dead (KD) form. Red arrows point to the place of injury. **(I)** Morphometric analysis (Average Length) of sLNv axonal projections where developmental overexpression of *faf* and Wnd RNAi has been specifically induced in the sLNvs, uncovering a Faf-Wnd gene interaction. Axonal outgrowth is measured in μm. **(J)** Percentage of brains showing at least one regenerated axonal sprout 4 days after injury (Capacity of regrowth), where overexpression of *wnd* and Wnd KD has been specifically induced in the sLNvs. Note that **A,A**′ are the same as in Figures 4A,A′, and **F,F**′ the same as in Figures 5B,B′. Genotype of flies in **(A,A**′**)** is PDF-Gal4, UAS-GFP/+; PDF-Gal4, UAS-2x eGFP/+; UAS-Faf/+, in **(B,B')** is PDF-Gal4, UAS-GFP/+; PDF-Gal4, UAS-2x eGFP/+; UAS-Faf/Wnd RNAi;, in (**C, C'** and **G,G**′) is PDF-Gal4, UAS-GFP/+; PDF-Gal4, UAS-2x eGFP/UAS-Wnd E;, in (**D,D**′ and **H,H**′) is PDF-Gal4, UAS-GFP/+; PDF-Gal4, UAS-2x eGFP/+; UAS-GFP,UAS-Wnd KD, in **(E,E**′**)** is PDF-Gal4, UAS-GFP/+; /UAS-Wnd E/ Dscam RNAi;, in **(F,F**′**)** is PDF-Gal4, UAS-GFP/+; PDF-Gal4, UAS-2x eGFP/Dscam RNAi;. Dotted insets have been zoomed in to better illustrate the diverse axonal phenotypes obtained. Scale bars are 20 μm.

Supplementary Figure 4 Dscam containing the TM1 domain localizes to both axonal projections as well as cell bodies and dendrites of sLNvs. **(A,B)** A Dscam form containing the TM1 domain (Dscam1-1.34.31.1 HA) localizes to both dendrites and axonal projections, and to the cell bodies. An antibody against the pigment dispersing factor hormone (PDF) specifically stains PDF neurons **(A,B)**. Dscam expression pattern was visualized using an antibody against HA **(A**′**,B**′**)**. Genotype of flies is PDF-Gal4, UAS-GFP/+; PDF-Gal4, UAS-2x eGFP/+; UAS-Dscam1-1.34.31.1.HA. Scale bars are 30 μm.

Supplementary Table 1 Candidate genes tested in the genetic screen for axonal outgrowth in development.

Supplementary Table 2 Number of samples across the various experiments.

## References

[B1] Ambrozkiewicz M. C. Kawabe H. (2015). HECT-type E3 ubiquitin ligases in nerve cell development and synapse physiology. FEBS Lett. 589, 1635–1643. 10.1016/j.febslet.2015.05.009 25979171

[B2] Arthur-Farraj P. J. Latouche M. Wilton D. K. Quintes S. Chabrol E. Banerjee A.. (2012). c-Jun reprograms Schwann cells of injured nerves to generate a repair cell essential for regeneration. Neuron 75, 633–647. 10.1016/j.neuron.2012.06.021 22920255 PMC3657176

[B3] Ayaz D. Leyssen M. Koch M. Yan J. Srahna M. Sheeba V.. (2008). Axonal injury and regeneration in the adult brain of *Drosophila*. J. Neurosci. 28, 6010–6021. 10.1523/JNEUROSCI.0101-08.2008 18524906 PMC2693324

[B4] Belin S. Nawabi H. Wang C. Tang S. Latremoliere A. Warren P.. (2015). Injury-induced decline of intrinsic regenerative ability revealed by quantitative proteomics. Neuron 86, 1000–1014. 10.1016/j.neuron.2015.03.060 25937169 PMC4551425

[B5] Bischof J. Maeda R. K. Hediger M. Karch F. Basler K. (2007). An optimized transgenesis system for *Drosophila* using germ-line-specific phiC31 integrases. Proc. Natl. Acad. Sci. U.S.A. 104, 3312–3317. 10.1073/pnas.0611511104 17360644 PMC1805588

[B6] Blackmore M. G. Wang Z. Lerch J. K. Motti D. Zhang Y. P. Shields C. B.. (2012). Kruppel-like Factor 7 engineered for transcriptional activation promotes axon regeneration in the adult corticospinal tract. Proc. Natl. Acad. Sci. U.S.A. 109, 7517–7522. 10.1073/pnas.1120684109 22529377 PMC3358880

[B7] Brand A. H. Perrimon N. (1993). Targeted gene expression as a means of altering cell fates and generating dominant phenotypes. Development 118, 401–415. 8223268 10.1242/dev.118.2.401

[B8] Byrne A. B. Walradt T. Gardner K. E. Hubbert A. Reinke V. Hammarlund M. (2014). Insulin/IGF1 signaling inhibits age-dependent axon regeneration. Neuron 81, 561–573. 10.1016/j.neuron.2013.11.019 24440228 PMC3924874

[B9] Cafferty W. B. Duffy P. Huebner E. Strittmatter S. M. (2010). MAG and OMgp synergize with Nogo-A to restrict axonal growth and neurological recovery after spinal cord trauma. J. Neurosci. 30, 6825–6837. 10.1523/JNEUROSCI.6239-09.2010 20484625 PMC2883258

[B10] Cai D. Qiu J. Cao Z. McAtee M. Bregman B. S. Filbin M. T. (2001). Neuronal cyclic AMP controls the developmental loss in ability of axons to regenerate. J. Neurosci. 21, 4731–4739. 11425900 10.1523/JNEUROSCI.21-13-04731.2001 PMC6762375

[B11] Chen L. Wang Z. Ghosh-Roy A. Hubert T. Yan D. O'Rourke S.. (2011). Axon regeneration pathways identified by systematic genetic screening in C. elegans. Neuron 71, 1043–1057. 10.1016/j.neuron.2011.07.009 21943602 PMC3183436

[B12] Chen X. Fischer J. A. (2000). *In vivo* Structure/Function analysis of the *Drosophila* fat facets deubiquitinating enzyme gene. Genetics 156, 1829–1836. 11102377 10.1093/genetics/156.4.1829 PMC1461389

[B13] Chen X. Overstreet E. Wood S. A. Fischer J. A. (2000). On the conservation of function of the *Drosophila* fat facets deubiquitinating enzyme and Fam, its mouse homolog. Dev. Genes Evol. 210, 603–610. 10.1007/s004270000109 11151297

[B14] Collins C. A. Wairkar Y. P. Johnson S. L. DiAntonio A. (2006). Highwire restrains synaptic growth by attenuating a MAP kinase signal. Neuron 51, 57–69. 10.1016/j.neuron.2006.05.026 16815332

[B15] DiAntonio A. Haghighi A. P. Portman S. L. Lee J. D. Amaranto A. M. Goodman C. S. (2001). Ubiquitination-dependent mechanisms regulate synaptic growth and function. Nature 412, 449–452. 10.1038/35086595 11473321

[B16] Duan X. Qiao M. Bei F. Kim I. J. He Z. Sanes J. R. (2015). Subtype-specific regeneration of retinal ganglion cells following axotomy: effects of osteopontin and mTOR signaling. Neuron 85, 1244–1256. 10.1016/j.neuron.2015.02.017 25754821 PMC4391013

[B17] El Bejjani R. Hammarlund M. (2012). Notch signaling inhibits axon regeneration. Neuron 73, 268–278. 10.1016/j.neuron.2011.11.017 22284182 PMC3690129

[B18] Fang Y. Bonini N. M. (2012). Axon degeneration and regeneration: insights from *Drosophila* models of nerve injury. Annu. Rev. Cell Dev. Biol. 28, 575–597. 10.1146/annurev-cellbio-101011-155836 22831639

[B19] Fang Y. Soares L. Teng X. Geary M. Bonini N. M. (2012). A novel *Drosophila* model of nerve injury reveals an essential role of Nmnat in maintaining axonal integrity. Curr. Biol. 22, 590–595. 10.1016/j.cub.2012.01.065 22425156 PMC3347919

[B20] Gabel C. V. Antoine F. Chuang C. F. Samuel A. D. Chang C. (2008). Distinct cellular and molecular mechanisms mediate initial axon development and adult-stage axon regeneration in *C. elegans*. Development 135, 1129–1136. 10.1242/dev.013995 18296652

[B21] Goldberg J. L. Klassen M. P. Hua Y. Barres B. A. (2002). Amacrine-signaled loss of intrinsic axon growth ability by retinal ganglion cells. Science 296, 1860–1864. 10.1126/science.1068428 12052959

[B22] Hao Y. Frey E. Yoon C. Wong H. Nestorovski D. Holzman L. B.. (2016). An evolutionarily conserved mechanism for cAMP elicited axonal regeneration involves direct activation of the dual leucine zipper kinase DLK. Elife 5:e14048. 10.7554/eLife.14048 27268300 PMC4896747

[B23] Harel N. Y. Strittmatter S. M. (2006). Can regenerating axons recapitulate developmental guidance during recovery from spinal cord injury? Nat. Rev. Neurosci. 7, 603–616. 10.1038/nrn1957 16858389 PMC2288666

[B24] Hassan B. A. Bermingham N. A. He Y. Sun Y. Jan Y. N. Zoghbi H. Y. (2000). atonal regulates neurite arborization but does not act as a proneural gene in the *Drosophila* brain. Neuron 25, 549–561. 10.1016/S0896-6273(00)81059-4 10774724

[B25] He H. Kise Y. Izadifar A. Urwyler O. Ayaz D. Parthasarthy A.. (2014). Cell-intrinsic requirement of Dscam1 isoform diversity for axon collateral formation. Science 344, 1182–1186. 10.1126/science.1251852 24831526

[B26] Helfrich-Förster C. Yoshii T. Wülbeck C. Grieshaber E. Rieger D. Bachleitner W.. (2007). The lateral and dorsal neurons of *Drosophila melanogaster*: new insights about their morphology and function. Cold Spring Harb. Symp. Quant. Biol. 72, 517–525. 10.1101/sqb.2007.72.063 18419311

[B27] Homan C. C. Kumar R. Nguyen L. S. Haan E. Raymond F. L. Abidi F.. (2014). Mutations in USP9X are associated with X-linked intellectual disability and disrupt neuronal cell migration and growth. Am. J. Hum. Genet. 94, 470–478. 10.1016/j.ajhg.2014.02.004 24607389 PMC3951929

[B28] Huang Y. Baker R. T. Fischer-Vize J. A. (1995). Control of cell fate by a deubiquitinating enzyme encoded by the fat facets gene. Science 270, 1828–1831. 10.1126/science.270.5243.1828 8525378

[B29] Hughes M. E. Bortnick R. Tsubouchi A. Bäumer P. Kondo M. Uemura T.. (2007). Homophilic Dscam interactions control complex dendrite morphogenesis. Neuron 54, 417–427. 10.1016/j.neuron.2007.04.013 17481395 PMC1963440

[B30] Itoh A. Horiuchi M. Bannerman P. Pleasure D. Itoh T. (2009). Impaired regenerative response of primary sensory neurons in ZPK/DLK gene-trap mice. Biochem. Biophys. Res. Commun. 383, 258–262. 10.1016/j.bbrc.2009.04.009 19358824

[B31] Kaplan A. Ong Tone S. Fournier A. E. (2015). Extrinsic and intrinsic regulation of axon regeneration at a crossroads. Front. Mol. Neurosci. 8:27. 10.3389/fnmol.2015.00027 26136657 PMC4470051

[B32] Kato K. Forero M. G. Fenton J. C. Hidalgo A. (2011). The glial regenerative response to central nervous system injury is enabled by pros-notch and pros-NFkappaB feedback. PLoS Biol. 9:e1001133. 10.1371/journal.pbio.1001133 21912512 PMC3166069

[B33] Kim J. H. Wang X. Coolon R. Ye B. (2013). Dscam expression levels determine presynaptic arbor sizes in *Drosophila* sensory neurons. Neuron 78, 827–838. 10.1016/j.neuron.2013.05.020 23764288 PMC3709448

[B34] Klueg K. M. Alvarado D. Muskavitch M. A. Duffy J. B. (2002). Creation of a GAL4/UAS-coupled inducible gene expression system for use in *Drosophila* cultured cell lines. Genesis 34, 119–122. 10.1002/gene.10148 12324964

[B35] Koch M. H. B. A. (2012). Out with the Brain: *Drosophila* Whole-Brain Explant Culture. The making and un-making of neuronal circuits in Drosophila, in Neuromethods Series, ed Hassan B. A. (Springer; Humana Press), 261–268.

[B36] Lee J. K. Geoffroy C. G. Chan A. F. Tolentino K. E. Crawford M. J. Leal M. A.. (2010). Assessing spinal axon regeneration and sprouting in Nogo-, MAG-, and OMgp-deficient mice. Neuron 66, 663–670. 10.1016/j.neuron.2010.05.002 20547125 PMC2896331

[B37] Leyssen M. Ayaz D. Hébert S. S. Reeve S. De Strooper B. Hassan B. A. (2005). Amyloid precursor protein promotes post-developmental neurite arborization in the *Drosophila* brain. EMBO J. 24, 2944–2955. 10.1038/sj.emboj.7600757 16052209 PMC1187942

[B38] Li C. Hisamoto N. Nix P. Kanao S. Mizuno T. Bastiani M.. (2012). The growth factor SVH-1 regulates axon regeneration in C. elegans via the JNK MAPK cascade. Nat. Neurosci. 15, 551–557. 10.1038/nn.3052 22388962

[B39] Liu K. Lu Y. Lee J. K. Samara R. Willenberg R. Sears-Kraxberger I.. (2010). PTEN deletion enhances the regenerative ability of adult corticospinal neurons. Nat. Neurosci. 13, 1075–1081. 10.1038/nn.2603 20694004 PMC2928871

[B40] Liu K. Tedeschi A. Park K. K. He Z. (2011). Neuronal intrinsic mechanisms of axon regeneration. Annu. Rev. Neurosci. 34, 131–152. 10.1146/annurev-neuro-061010-113723 21438684

[B41] MacDonald J. M. Beach M. G. Porpiglia E. Sheehan A. E. Watts R. J. Freeman M. R. (2006). The *Drosophila* cell corpse engulfment receptor Draper mediates glial clearance of severed axons. Neuron 50, 869–881. 10.1016/j.neuron.2006.04.028 16772169

[B42] Makwana M. Raivich G. (2005). Molecular mechanisms in successful peripheral regeneration. FEBS J. 272, 2628–2638. 10.1111/j.1742-4658.2005.04699.x 15943798

[B43] McCabe B. D. Hom S. Aberle H. Fetter R. D. Marques G. Haerry T. E.. (2004). Highwire regulates presynaptic BMP signaling essential for synaptic growth. Neuron 41, 891–905. 10.1016/S0896-6273(04)00073-X 15046722

[B44] Moore D. L. Blackmore M. G. Hu Y. Kaestner K. H. Bixby J. L. Lemmon V. P.. (2009). KLF family members regulate intrinsic axon regeneration ability. Science 326, 298–301. 10.1126/science.1175737 19815778 PMC2882032

[B45] Nix P. Hisamoto N. Matsumoto K. Bastiani M. (2011). Axon regeneration requires coordinate activation of p38 and JNK MAPK pathways. Proc. Natl. Acad. Sci. U.S.A. 108, 10738–10743. 10.1073/pnas.1104830108 21670305 PMC3127873

[B46] Overstreet E. Fitch E. Fischer J. A. (2004). Fat facets and Liquid facets promote Delta endocytosis and Delta signaling in the signaling cells. Development 131, 5355–5366. 10.1242/dev.01434 15469967

[B47] Peterson G. L. (1977). A simplification of the protein assay method of Lowry et al. which is more generally applicable. Anal. Biochem. 83, 346–356. 10.1016/0003-2697(77)90043-4 603028

[B48] Qu C. Li W. Shao Q. Dwyer T. Huang H. Yang T.. (2013). c-Jun N-terminal kinase 1 (JNK1) is required for coordination of netrin signaling in axon guidance. J. Biol. Chem. 288, 1883–1895. 10.1074/jbc.M112.417881 23223444 PMC3548497

[B49] Raivich G. Bohatschek M. Da Costa C. Iwata O. Galiano M. Hristova M.. (2004). The AP-1 transcription factor c-Jun is required for efficient axonal regeneration. Neuron 43, 57–67. 10.1016/j.neuron.2004.06.005 15233917

[B50] Raivich G. Makwana M. (2007). The making of successful axonal regeneration: genes, molecules and signal transduction pathways. Brain Res. Rev. 53, 287–311. 10.1016/j.brainresrev.2006.09.005 17079020

[B51] Schmucker D. Clemens J. C. Shu H. Worby C. A. Xiao J. Muda M.. (2000). *Drosophila* Dscam is an axon guidance receptor exhibiting extraordinary molecular diversity. Cell 101, 671–684. 10.1016/S0092-8674(00)80878-8 10892653

[B52] Shi L. Yu H. H. Yang J. S. Lee T. (2007). Specific *Drosophila* Dscam juxtamembrane variants control dendritic elaboration and axonal arborization. J. Neurosci. 27, 6723–6728. 10.1523/JNEUROSCI.1517-07.2007 17581959 PMC6672701

[B53] Shimizu I. Oppenheim R. W. O'Brien M. Shneiderman A. (1990). Anatomical and functional recovery following spinal cord transection in the chick embryo. J. Neurobiol. 21, 918–937. 10.1002/neu.480210609 2077104

[B54] Stegeman S. Jolly L. A. Premarathne S. Gecz J. Richards L. J. Mackay-Sim A.. (2013). Loss of *Usp9x* disrupts cortical architecture, hippocampal development and TGFβ-mediated axonogenesis. PLoS ONE 8:e68287. 10.1371/journal.pone.0068287 23861879 PMC3702552

[B55] Sun F. Park K. K. Belin S. Wang D. Lu T. Chen G.. (2011). Sustained axon regeneration induced by co-deletion of PTEN and SOCS3. Nature 480, 372–375. 10.1038/nature10594 22056987 PMC3240702

[B56] Valakh V. Frey E. Babetto E. Walker L. J. DiAntonio A. (2015). Cytoskeletal disruption activates the DLK/JNK pathway, which promotes axonal regeneration and mimics a preconditioning injury. Neurobiol. Dis. 77, 13–25. 10.1016/j.nbd.2015.02.014 25726747 PMC4402261

[B57] Watkins T. A. Wang B. Huntwork-Rodriguez S. Yang J. Jiang Z. Eastham-Anderson J.. (2013). DLK initiates a transcriptional program that couples apoptotic and regenerative responses to axonal injury. Proc. Natl. Acad. Sci. U.S.A. 110, 4039–4044. 10.1073/pnas.1211074110 23431164 PMC3593899

[B58] Watson F. L. Püttmann-Holgado R. Thomas F. Lamar D. L. Hughes M. Kondo M.. (2005). Extensive diversity of Ig-superfamily proteins in the immune system of insects. Science 309, 1874–1878. 10.1126/science.1116887 16109846

[B59] Wood S. A. Pascoe W. S. Ru K. Yamada T. Hirchenhain J. Kemler R.. (1997). Cloning and expression analysis of a novel mouse gene with sequence similarity to the *Drosophila* fat facets gene. Mech. Dev. 63, 29–38. 10.1016/S0925-4773(97)00672-2 9178254

[B60] Wu C. Daniels R. W. DiAntonio A. (2007). DFsn collaborates with Highwire to down-regulate the Wallenda/DLK kinase and restrain synaptic terminal growth. Neural Dev. 12, 16 10.1186/1749-8104-2-16 PMC2031890 17697379

[B61] Yan D. Wu Z. Chisholm A. D. Jin Y. (2009). The DLK-1 kinase promotes mRNA stability and local translation in *C*. elegans synapses and axon regeneration. Cell 138, 1005–1018. 10.1016/j.cell.2009.06.023 19737525 PMC2772821

[B62] Yanik M. F. Cinar H. Cinar H. N. Chisholm A. D. Jin Y. Ben-Yakar A. (2004). Neurosurgery: functional regeneration after laser axotomy. Nature 432, 822. 10.1038/432822a 15602545

[B63] Yaniv S. P. Issman-Zecharya N. Oren-Suissa M. Podbilewicz B. Schuldiner O. (2012). Axon regrowth during development and regeneration following injury share molecular mechanisms. Curr. Biol. 22, 1774–1782. 10.1016/j.cub.2012.07.044 22921367

