# Supplementary figures and images for: A Fat-Facets-Dscam1-JNK Pathway Enhances Axonal Growth in Development and after Injury
Source: Front Cell Neurosci. 2018 Feb 8;11:416. doi: 10.3389/fncel.2017.00416 (PMC5809495; doi:10.3389/fncel.2017.00416)

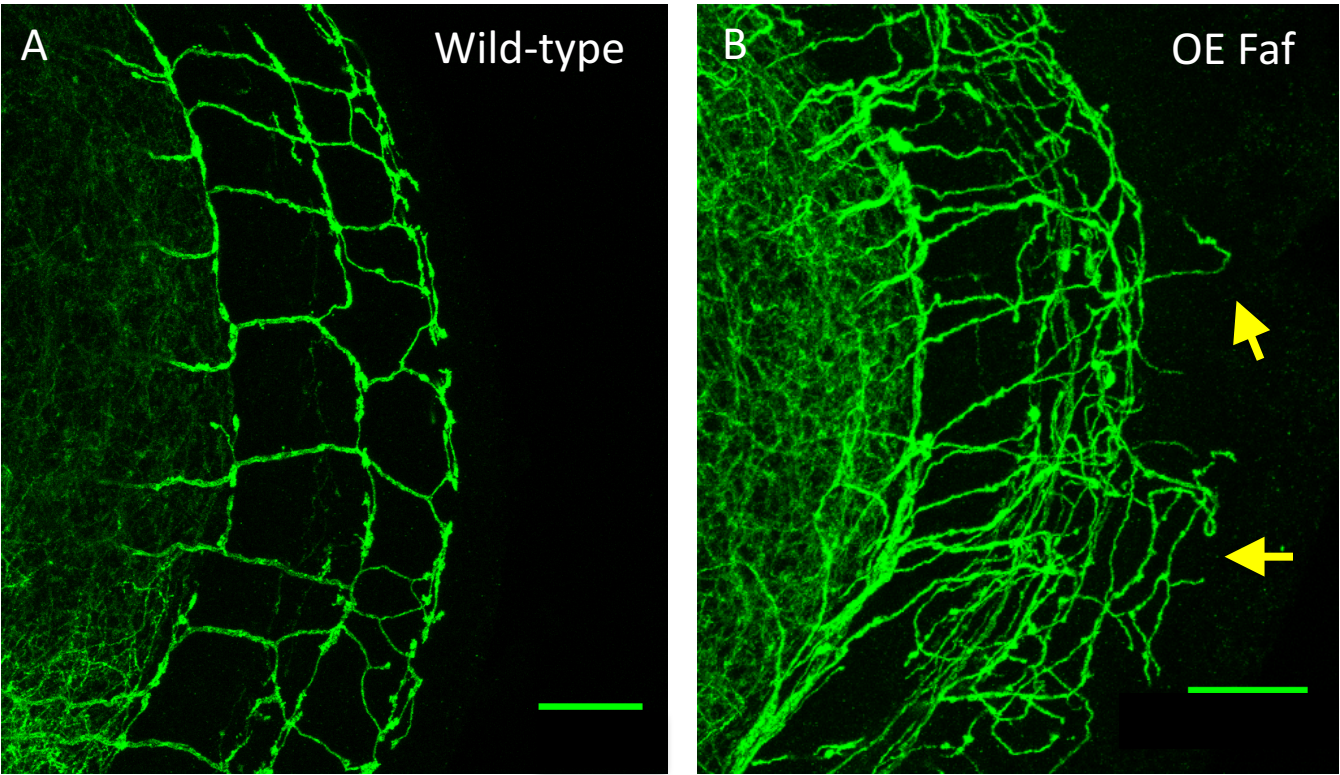

Supplementary Figure 1 (Related to Figure 3)

Supplement: Supplementary Figure 1 — Faf gain of function promotes axonal growth in a distinct neuronal population. (A,B) Overexpression of faf specifically in the Dorsal Cluster Neurons (DCNs) results in increased axonal growth (yellow arrows) (B), in comparison to wild-type flies (A). Genotype of flies in (A,A′) is;UAS-GFP;ato-Gal4 14a, in (B,B′);UAS-GFP;ato-Gal4 14a/UAS-Faf. Scale bars are 20 μm. [file Image1.pdf]

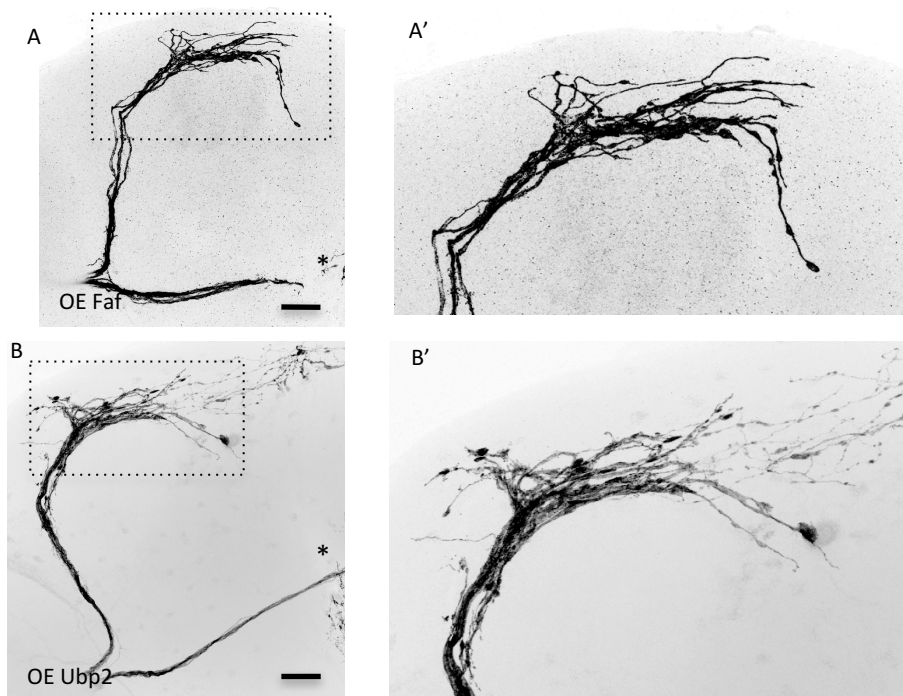

Supplementary Figure 2 (Related to Figures 2 and 4)

Supplement: Supplementary Figure 2 — Ubp2 gain of function also promotes axonal growth. (A,B) Overexpression of the yeast homolog of Faf, Ubp2, which shows conservation of the enzymatic domain, also results in increased axonal growth (B), in a similar manner to Faf overexpression (A). Note that (B,B′) are the same as in Figures 4A,A'. Genotype of flies in (A,A') is PDF-Gal4, UAS-GFP/+; PDF-Gal4, UAS-2x eGFP/+; UAS-Faf/+; in ((B,B′) is PDF-Gal4, UAS-GFP/+; PDF-Gal4, UAS-2x eGFP/UAS-Ubp2. Dotted insets have been zoomed in to better illustrate the diverse axonal phenotypes obtained. Scale bars are 30 μm. [file Image2.pdf]

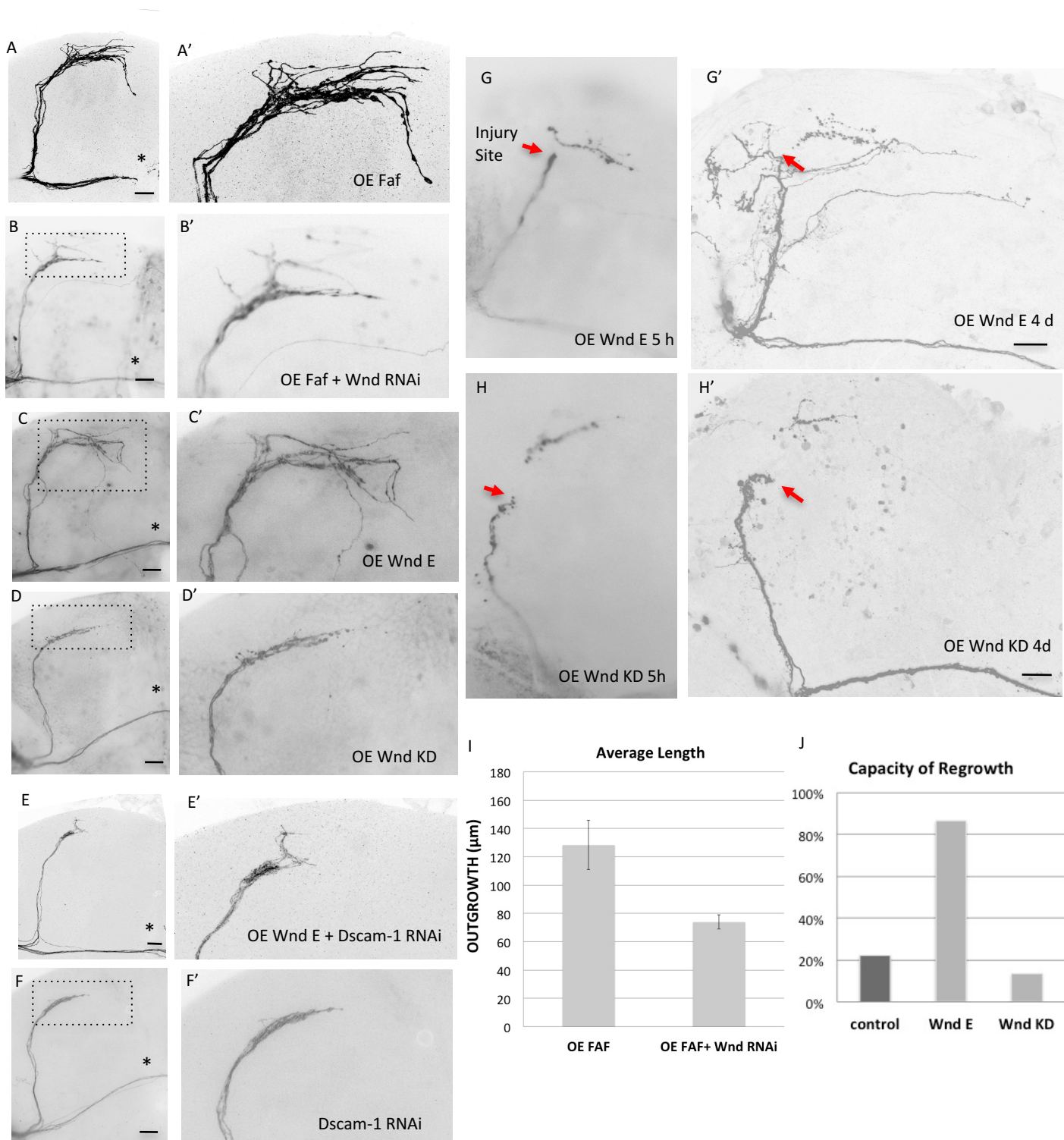

Supplementary Figure 3 (Related to Figures 4 and 5)

Supplement: Supplementary Figure 3 — Wallenda promotes growth in development and after injury and is required for Faf-induced growth. (A,B) Representative images of sLNv axonal arborization demonstrating that knock-down of wnd inhibits Faf-induced outgrowth. (C,D) Representative images of sLNv axonal arborization in adult flies where developmental overexpression of wnd (C,C′), but not of a kinase dead form (D,D′) in the sLNvs induces axonal growth similar to the one induced by Faf (A,A′). (E,F) Representative images of sLNv axonal arborization demonstrating that knock-down of Dscam1 inhibits Wnd-induced outgrowth (E,E′) and results in a phenotype that resembles knock-down of Dscam1 on its own (F,F′). (G,H) Overexpression of wnd in the sLNVs (G,G′), but not of Wnd KD (H,H′) induces axonal regrowth 4 days after injury. Five hour after injury timepoints (G,H) have been included to better illustrate the regenerative ability of Wnd, but not of its kinase dead (KD) form. Red arrows point to the place of injury. (I) Morphometric analysis (Average Length) of sLNv axonal projections where developmental overexpression of faf and Wnd RNAi has been specifically induced in the sLNvs, uncovering a Faf-Wnd gene interaction. Axonal outgrowth is measured in μm. (J) Percentage of brains showing at least one regenerated axonal sprout 4 days after injury (Capacity of regrowth), where overexpression of wnd and Wnd KD has been specifically induced in the sLNvs. Note that A,A′ are the same as in Figures 4A,A′, and F,F′ the same as in Figures 5B,B′. Genotype of flies in (A,A′) is PDF-Gal4, UAS-GFP/+; PDF-Gal4, UAS-2x eGFP/+; UAS-Faf/+, in (B,B') is PDF-Gal4, UAS-GFP/+; PDF-Gal4, UAS-2x eGFP/+; UAS-Faf/Wnd RNAi;, in (C, C' and G,G′) is PDF-Gal4, UAS-GFP/+; PDF-Gal4, UAS-2x eGFP/UAS-Wnd E;, in (D,D′ and H,H′) is PDF-Gal4, UAS-GFP/+; PDF-Gal4, UAS-2x eGFP/+; UAS-GFP,UAS-Wnd KD, in (E,E′) is PDF-Gal4, UAS-GFP/+; /UAS-Wnd E/ Dscam RNAi;, in (F,F′) is PDF-Gal4, UAS-GFP/+; PDF-Gal4, UAS-2x eGFP/Dscam RNAi;. Dotted insets have been z [file Image3.pdf]

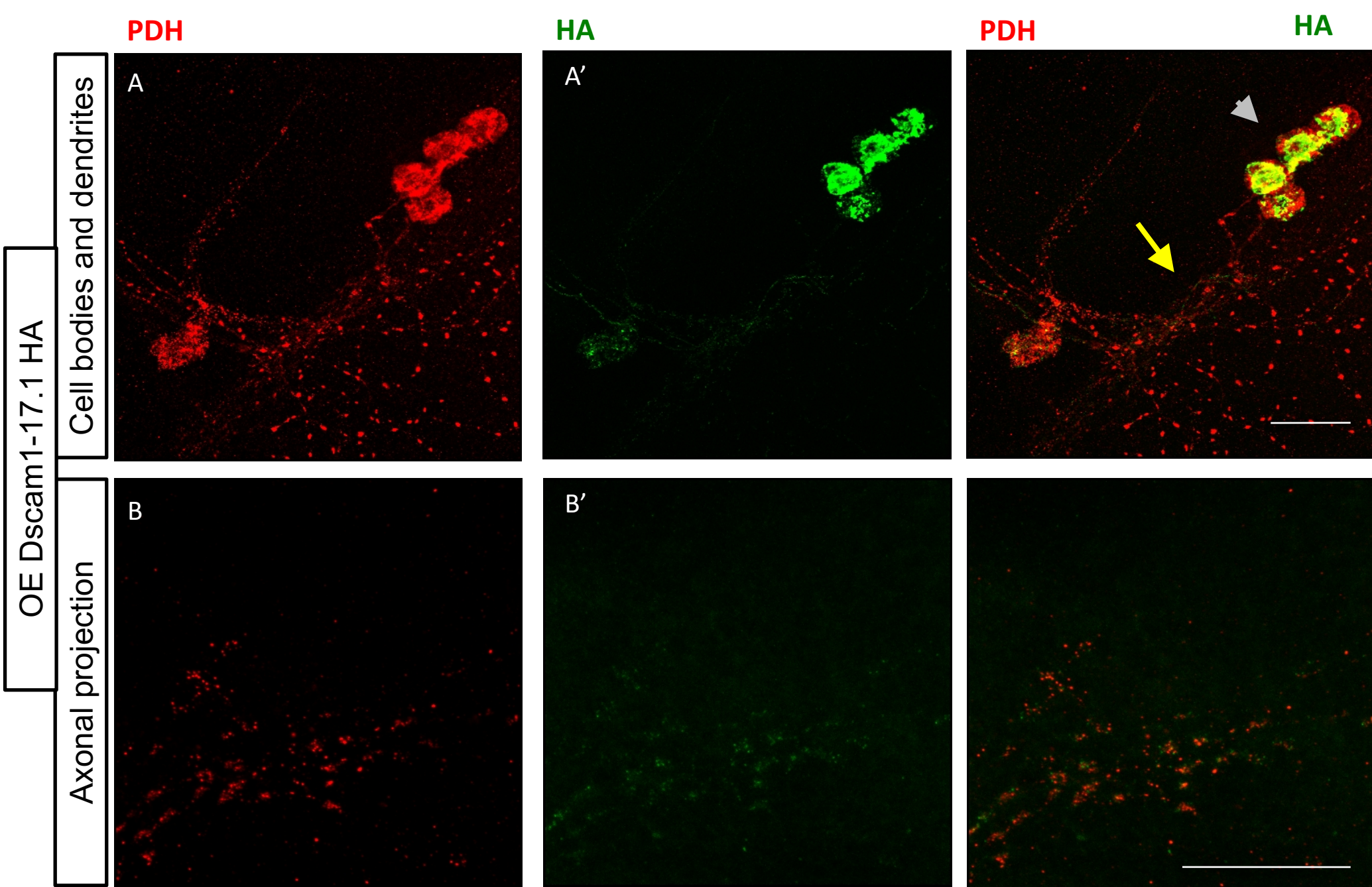

Supplementary Figure 4 (Related to Figure 5)

Supplement: Supplementary Figure 4 — Dscam containing the TM1 domain localizes to both axonal projections as well as cell bodies and dendrites of sLNvs. (A,B) A Dscam form containing the TM1 domain (Dscam1-1.34.31.1 HA) localizes to both dendrites and axonal projections, and to the cell bodies. An antibody against the pigment dispersing factor hormone (PDF) specifically stains PDF neurons (A,B). Dscam expression pattern was visualized using an antibody against HA (A′,B′). Genotype of flies is PDF-Gal4, UAS-GFP/+; PDF-Gal4, UAS-2x eGFP/+; UAS-Dscam1-1.34.31.1.HA. Scale bars are 30 μm. [file Image4.pdf]
